# Measurement invariance within and between individuals: a distinct problem in testing the equivalence of intra- and inter-individual model structures

**DOI:** 10.3389/fpsyg.2014.00883

**Published:** 2014-09-19

**Authors:** Janne Adolf, Noémi K. Schuurman, Peter Borkenau, Denny Borsboom, Conor V. Dolan

**Affiliations:** ^1^Center for Lifespan Psychology, Max Planck Institute for Human DevelopmentBerlin, Germany; ^2^Department of Methodology and Statistics, Faculty of Social and Behavioral Sciences, Utrecht UniversityUtrecht, Netherlands; ^3^Personality and Diagnostics Group, Department of Psychology, Faculty of Philosophy I, Martin-Luther-University Halle-WittenbergHalle, Germany; ^4^Psychological Methods Group, Department of Psychology, Faculty of Social and Behavioral Sciences, University of AmsterdamAmsterdam, Netherlands; ^5^Department of Biological Psychology, Faculty of Psychology and Education, Free University of AmsterdamAmsterdam, Netherlands

**Keywords:** measurement invariance, ergodicity, state-space modeling, latent variables, intra-individual level of analysis

## Abstract

We address the question of equivalence between modeling results obtained on intra-individual and inter-individual levels of psychometric analysis. Our focus is on the concept of measurement invariance and the role it may play in this context. We discuss this in general against the background of the latent variable paradigm, complemented by an operational demonstration in terms of a linear state-space model, i.e., a time series model with latent variables. Implemented in a multiple-occasion and multiple-subject setting, the model simultaneously accounts for intra-individual and inter-individual differences. We consider the conditions—in terms of invariance constraints—under which modeling results are generalizable (a) over time within subjects, (b) over subjects within occasions, and (c) over time and subjects simultaneously thus implying an equivalence-relationship between both dimensions. Since we distinguish the measurement model from the structural model governing relations between the latent variables of interest, we decompose the invariance constraints into those that involve structural parameters and those that involve measurement parameters and relate to measurement invariance. Within the resulting taxonomy of models, we show that, under the condition of measurement invariance over time and subjects, there exists a form of structural equivalence between levels of analysis that is distinct from full structural equivalence, i.e., ergodicity. We demonstrate how measurement invariance between and within subjects can be tested in the context of high-frequency repeated measures in personality research. Finally, we relate problems of measurement variance to problems of non-ergodicity as currently discussed and approached in the literature.

## Introduction

Population heterogeneity exists when multiple distinct statistical models are required to adequately describe a population (Muthén, 1989). Statistical approaches to investigate and accommodate heterogeneity include, for instance, multi-group modeling (e.g., Jöreskog, 1971; Muthén, 1989), multi-level modeling (e.g., Hox, 2002), and structural equation mixture modeling (e.g., Dolan, 2009). In each of these modeling approaches a heterogeneous population is stratified into subpopulations whose members adhere to the same models and differences within are separated from differences between subpopulations (Muthén, 1989). But how small is the smallest subgroup? One could think of a scenario in which breaking up a heterogeneous population into ever smaller subpopulations leads to the smallest subpopulation that is empirically realizable. This is the individual person (Millsap, 2011). Consider, for instance, the five-factor-model (FFM) which states that the dimensions Extraversion, Neuroticism, Agreeableness, Conscientiousness and Openness to Experience are the major sources of inter-individual differences in personality (McCrae and John, 1992). A researcher studying population heterogeneity can now well question, whether the FFM is generally interpretable in the sense that it holds for each individual member of the overall population by addressing “universal” determinants of human behavior (Hamaker et al., 2005).

Questions of this kind have indeed been posed recently and have been addressed by means of single subject (*N* = 1) modeling based on the analysis of repeated measurements over occasions (Cattell, 1952; Gregson, 1983; Molenaar, 1985). By contrasting intra-individual with inter-individual difference data, it has been shown that inter-individual modeling results do usually not generalize to the level of the individual. Rather, individual specifics, which remain undetected in standard large sample modeling techniques, seem to be the rule, not the exception (e.g., Molenaar et al., 2003; Molenaar, 2004; Hamaker et al., 2005, 2007; Kelderman and Molenaar, 2007; Molenaar and Campbell, 2009; Schmiedek et al., 2009; Brose et al., 2010, 2014; Nesselroade, 2010). The increasing interest in individual modeling techniques therefore emphasizes the conceptual continuity between approaches to heterogeneous populations and to the individual. Explicitly stated, single subject modeling accommodates population heterogeneity in its most extreme sense as it does not necessarily involve the generalization of results to other individuals or subpopulations of individuals. Each individual can thus potentially represent a system that is quantitatively or qualitatively unique (Molenaar, 2004).

We have so far conceived of heterogeneity as heterogeneity between individuals, but one may just as well conceive of heterogeneity as heterogeneity within individuals. That is, an individual's system characteristics may display (higher order) stability or variability over time (Molenaar, 2004). To illustrate this, suppose a researcher aims at describing a person with respect to a certain attribute over time. One may now think of an intra-individual distribution of states rather than of a single trait score. Considered over a representative set of situations, this distribution may have relatively stable characteristics over time, e.g., stable mean and variance. These may then be used to differentiate among people and may thus themselves be regarded as personality characteristics (Fleeson, 2001; Hamaker et al., 2007). However, also within individuals, homogeneity cannot be taken for granted but constitutes a (restrictedly) testable assumption. Similarly to questioning to what extent population models generalize to individual population members, one could question to what extent an individual time series model generalizes to (subsets of) single occasions.

The reorientation toward the individual in differential psychology has been motivated by and motivates an integrative consideration of the within- and the between-subject perspective. It therefore provides an optimal setting to address the following guiding questions: Under what conditions are modeling results generalizable (a) over occasions within subjects, (b) over subjects within occasions, and (c) over occasions and subjects simultaneously? Question (c) refers to the conditions that establish a systematic relationship, i.e., equivalence between the structure of intra- and the structure of inter-individual data (given large N and T). Borrowing terminology from statistical mechanics, this situation is termed *ergodicity* in the psychometric literature (e.g., Molenaar et al., 2003; Molenaar, 2004; Molenaar and Campbell, 2009). In the present context, ergodicity is referred to as a situation in which the statistical behavior of a time series observed for a single subject is the same as the statistical behavior of a sample of multiple subjects, obtained at a few occasions (i.e., the definition of an ergodic process according to Molenaar, 2004, p. 208).

Psychological attributes, however, are often represented as latent variables, the study of which requires psychometric measurement. In the context of latent variable modeling the conditions for an ergodic process decompose into invariance constraints on the structural part of the model and invariance constraints on the measurement model. The latter constraints relate to the concept of measurement invariance (MI; Mellenbergh, 1989; Meredith, 1993; Millsap, 2011). In this paper, we discuss how MI ties into the integrated within- and between-subject context. Specifically, we focus on how the concept is to be considered when one is interested in investigating the generalizability of latent variable modeling results along the dimensions time and subject.

The outline of the paper is as follows. Based on the definition as provided by Mellenbergh (1989), we elaborate on MI in the between- and within-subject context, in general terms and operationally in the linear factor model which lends itself well to integrated modeling, i.e., simultaneous modeling of intra-and inter-individual differences. We then proceed to address our guiding questions using a bottom-up approach. That is, in a multiple-subject, multiple-occasion setting, we set up a linear multi-subject latent variable time series model that accounts for intra-individual and inter-individual variability and we implement the model constraints that imply generalizability of results along the dimensions time and subject. We consider these constraints separately at the level of the measurement process and at the level of the latent psychological process. The result is a taxonomy of differently restrictive models ranging from full heterogeneity to full homogeneity between and within individuals. It can be considered a taxonomy of problems^1^ a researcher will potentially face when simultaneously modeling intra- and inter-individual variation. We show that MI holding simultaneously over time and subject can be interpreted as constituting a mode of structural equivalence between the intra- and the inter-individual level of analysis that is distinct from full structural equivalence. Using a real data illustration on intra-individual variability in the personality domain (Borkenau and Ostendorf, 1998), we show how researchers can test for MI over subjects and time. In the discussion, we reconsider the assumptions underlying MI testing and review alternative interpretations of and potential approaches to measurement variance within and between subjects.

## Measurement invariance between and within subjects

### General definition of measurement invariance

The present focus on MI is motivated by the latent variable paradigm which informs conceptual thinking in modern psychology (Bollen, 2002; Borsboom et al., 2003; Borsboom, 2008; Millsap, 2011). Although not directly observable, an attribute such as agreeableness can be conceptualized as manifesting in terms of observable behaviors or reportable attitudes, in this case along the interpersonal dimensions warmth, kindness, appreciation, and consideration (McCrae and John, 1992; Graziano and Tobin, 2009). However, inferences about latent variables on basis of observed indicators are subject to relatively large uncertainty (Borsboom, 2008). MI is one of the psychometric concepts addressing this uncertainty.

A general formal definition of MI in the latent variable paradigm was given by Mellenbergh (1989). Suppose we have a set of indicators ***Y*** that together form a psychometric instrument designed to measure a given latent variable *Z*, and suppose we have a variable *X*. MI of the indicators with respect to *X* is defined as independence of the indicators and *X* conditional on the latent variable, i.e.,

(1) f​(Y|Z=z)=f​(Y|Z=z, X=x)

for all values of *Z* and *X*, in which *f*( · ) denotes the probability distribution function. Under MI, any effect of *X* on the indicators is indirect, i.e., mediated through the latent variable (Lubke et al., 2003b). Consequently, significant differences in observed indicator scores are attributable to differences in the targeted latent variable (*Z*) across units selected on basis of *X*, e.g., across persons (e.g., Mellenbergh, 1989; Horn and McArdle, 1992; Lubke et al., 2003b; van der Sluis et al., 2006; Wicherts and Dolan, 2010; Millsap, 2011).

To illustrate this, imagine we attempted to measure agreeableness (*Z*) in a given sample using questionnaire ***Y***. Let *X* be the tendency to respond in a socially desirable manner (Paulhus and Reid, 1991; Holtgraves, 2004). If ***Y*** was measurement invariant with respect *X*, any two individuals from the sample having the same level of agreeableness would attain the same score on each item (apart from measurement error effects). Importantly, they would do so independent of their potentially different tendencies to respond in a socially desirable manner. ***Y*** would then be considered unbiased with respect to *X*. On the contrary, if ***Y*** was measurement variant or biased with respect to *X*, for instance due to item contents triggering socially desirable responding, differences in individual's responses would not necessarily be interpretable as differences in agreeableness. They may as well be interpretable as differences in socially desirable responding. Measurement variance or bias thus refers to a replicable difference in item scores which is not due to the targeted latent variable *Z* (Millsap, 2011). Meaningful comparisons in terms of the targeted latent variable are thus not guaranteed on basis of biased item scores (e.g., Dolan et al., 2004; Hamaker, 2007; Raykov et al., 2012).

Moreover, biased items can lead to biased estimates of parameters pertaining to the latent variable (Mellenbergh, 1989; Wicherts and Dolan, 2010). The interpretation of the latent variable is then rendered problematic. The converse argument would be that, if MI across persons selected on basis of *X* holds, the interpretation of the latent variable is the same across these persons (e.g., Mellenbergh, 1989; Horn and McArdle, 1992; Lubke et al., 2003a; Dolan et al., 2004; Borsboom and Dolan, 2007; Nesselroade et al., 2007; Wicherts and Dolan, 2010; Raykov et al., 2012). This notion of MI as *theoretical invariance*, as compared to the above notion of *unbiasedness*, can mainly be found for operationalizations of MI in the linear factor model. It is argued that the interpretation of the factor is determined by its relation to the observed indicators (the factor loadings) and that it is unlikely that different factors are related to a fixed set of indicators in exactly the same way (Lubke et al., 2003a).

Regardless of which interpretational notion is employed, in applying the concept of MI, one has to rely on premises which may appear more or less sensible depending on the context. We get back to this in more detail in the discussion.

### Conceptualization of measurement invariance between and within subjects

MI has been investigated extensively in the context of multi-group factor analysis, with groups defined by nominal between-subject variables, such as sex or ethnic background (e.g., van der Sluis et al., 2006; Wicherts and Dolan, 2010). Mellenbergh's definition, however, is a general one. It is neutral with respect to the nature and format of the potentially biasing variable, the indicator variables, and latent variables, and is thus independent of the psychometric model that relates the indicators to the latent variables (Mellenbergh, 1989; Meredith, 1993; Lubke et al., 2003a; Wicherts and Dolan, 2010). We can therefore draw two conclusions in the present context. First, Mellenbergh's definition should be equally applicable at the between-subject and at the within-subject level (Borsboom and Dolan, 2007). MI can also be considered with respect to time-varying variables relevant within subjects, such as mood or work pressure. For instance, a questionnaire supposed to assess intra-individual fluctuations in the state agreeableness over time may be biased with respect to mood. Then, a person's series of responses over time would reflect not only variations in the state agreeableness but additionally variations in mood. The second conclusion based on Mellenbergh's general definition is, that it is possible to take a more general perspective and consider MI with respect to subject and time (index) itself. This relates back to our introductory questions ^2^.

### Operationalization of measurement invariance between and within subjects

Mellenbergh's general MI definition gives rise to testable model constraints when implemented in the context of a concrete latent variable model. The latent variable modeling framework explicitly distinguishes between a (reflective) measurement model, in which the observed indicators are modeled as a function of the latent variables of psychological interest, and a structural model, which concerns the latent variables and their interrelationships. The linear factor model may be viewed as a proper measurement model in which multiple continuous indicators are linearly regressed upon a single continuous latent variable (e.g., Mellenbergh, 1994). In the linear factor model, MI has been associated with the constraints of strict factorial invariance (strict FI; Meredith, 1993) for the standard between-subject context. However, this measurement model features not only in structural equation modeling at the between-subject level (SEM) but also in state-space modeling of time series data at the within-subject level (SSM; Oud et al., 1990; Chow et al., 2010). We argue that strict FI should be equally applicable at the inter-individual and the intra-individual level. That is, strict FI over (subsets of) subjects within occasions, i.e., subject invariant measurement parameters such as factor loadings, intercepts and residual variances should almost certainly imply MI over subjects within occasions. In addition, strict FI over (subsets of) occasions or time within subjects, i.e., time-invariant measurement parameters, should almost certainly imply MI over time within subjects for the given sampling rate^3^.

## A bottom-up approach from full heterogeneity to ergodicity

### The baseline model

We now demonstrate the relation between ergodicity and MI in the context of linear stochastic time series models in state-space format (Harvey, 1989; Oud et al., 1990; Hamilton, 1994; Durbin and Koopman, 2001; Hamaker and Dolan, 2009; Chow et al., 2010). Such models primarily account for intra-individual variation over time. However, by specifying them within many subjects simultaneously we can extend them to multi-subject models. The conditions under which modeling results are generalizable over time, over subjects, and over time and subjects simultaneously may then be expressed in terms of specific invariance constraints. Furthermore, the state-space format incorporates a measurement model and a latent process model which allows distinguishing among constraints that apply to the measurement parameters and constraints that apply to latent parameters. In the following, subscript *i* and *t* refer to subject and discrete time, respectively. We assume equidistant measurement occasions throughout.

The latent process model is formulated as

(2) $$\eta_{i,\;t}=\alpha_{i,\;t}+{\text{B}}_{i,\;t}\eta_{i,\;t-1}+\;\zeta_{i,\;t}$$

where **η***_i, t_* is a *q* × 1 vector of latent variables, the states, which are regressed on themselves at the previous time point, **B***_i, t_* is a *q* × *q* matrix of latent regression parameters capturing the auto- and cross-lagged regression relationships among the states over time, and α*_i, t_* is a *q* × 1 vector of latent regression intercepts. The vector **ζ***_i, t_* is a *q* × 1 vector of latent residuals which are assumed to be multivariate normally distributed with mean zero and covariance matrix **Ψ***_i, t_*. The latent residuals are uncorrelated over time and uncorrelated with **η***_i, t_*_−1_. The model-implied mean vector of the latent states, **ν***_i, t_*, can be expressed as a function of **α***_i, t_*, **B***_i, t_*, and **ν***_i, t_*_−1_. The model-implied covariance-matrix of the latent states, **P***_i, t_*, can be expressed as a function of **B***_i, t_*, and **P***_i, t_*_−1_ and **Ψ***_i, t_*. Note that although the formal process is driven by a vector autoregressive process of first order, the actual psychological process needs not obey this structure. This so-called single lag structure renders the model fitting process technically convenient. However, any uni- or multivariate autoregressive moving average model can be accommodated (i.e., reformulated in terms of a first order vector autoregressive process) by extending the state vector by the relevant process components (e.g., Harvey, 1989; Hamaker and Dolan, 2009; Shumway and Stoffer, 2011).

The measurement model is formulated as

(3) $${\text{y}}_{i,\;t}=\tau_{i,\;t}+\Lambda_{i,\;t}\eta_{i,\;t}+\;\varepsilon_{i,\;t}$$

where **y***_i, t_* is a *p* × 1 vector of manifest indicators, **Λ***_i, t_* is a *p* × *q* matrix of factor loadings and **τ***_i, t_* is a *p* × 1 vector of measurement intercepts. The *p* × 1 vector **ε***_i, t_* contains measurement residuals, ideally measurement errors, which are assumed to be multivariate normally distributed with mean zero and covariance matrix **Θ***_i, t_*. The measurement residuals are uncorrelated over time and uncorrelated with **η***_i, t_* and **ζ***_i, t_*. Here, we additionally assume zero correlations among the measurement residuals, i.e., **Θ***_i, t_* is diagonal, satisfying the assumption of local independence. The model-implied mean vector of the indicators, **μ***_i, t_* can be expressed as a function of **τ***_i, t_*, **Λ***_i, t_*, and **ν***_i, t_*. The model-implied covariance-matrix of the indicators, **Σ***_i, t_*, can be expressed as a function of **Λ***_i, t_*, and **P***_i, t_* and **Θ***_i, t_*. As noted, this measurement model is equivalent to the linear factor model as it features in standard between-subject SEM (Oud et al., 1990; Chow et al., 2010).

**Figure 1:**
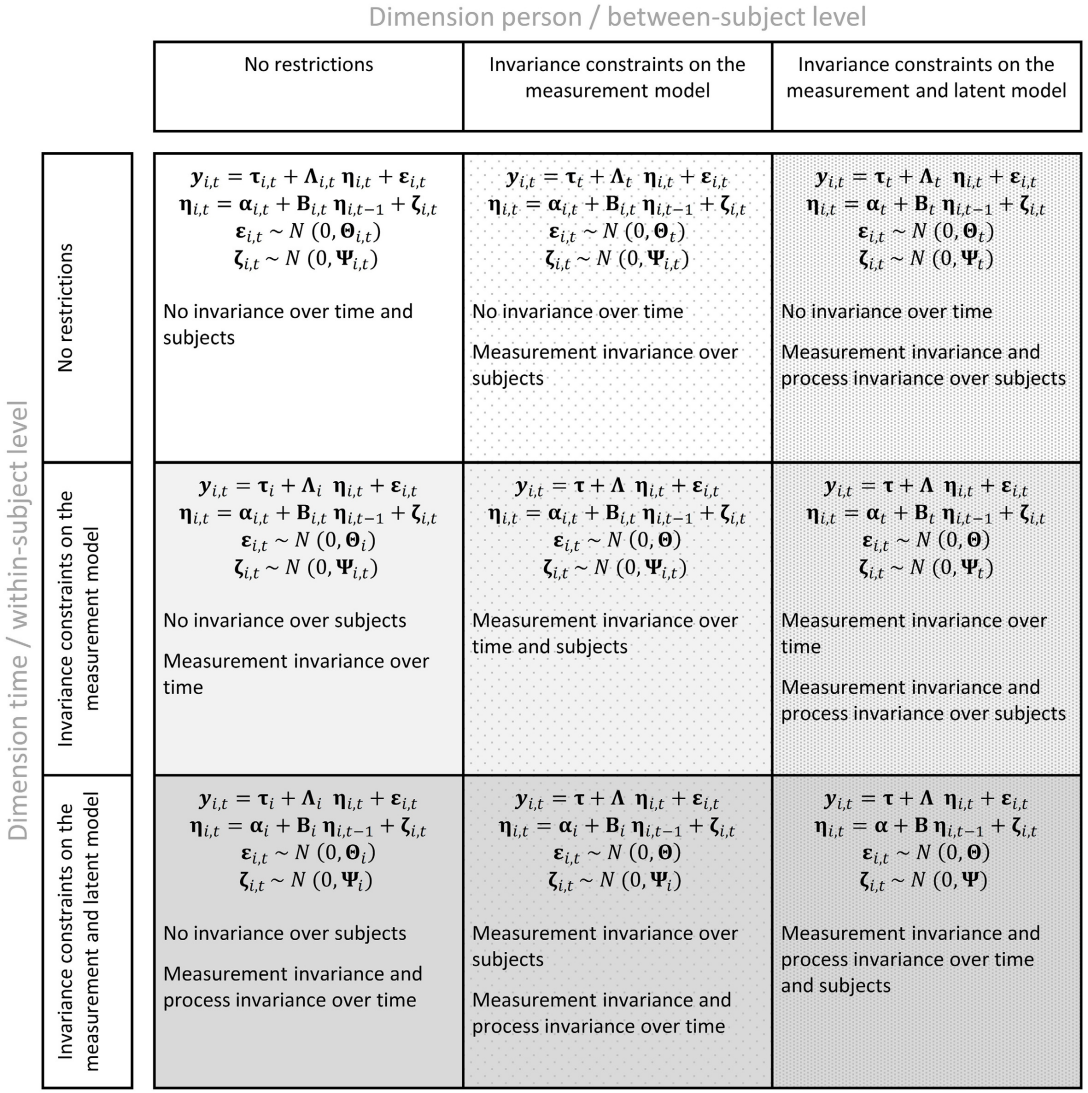
**Model taxonomy in terms of model equations and verbalized form**.

The model in Equations (2) and (3) is our baseline model. Note that the model is completely unrestricted with respect to time and subject, meaning that all model parameters can vary in value over time and subjects, but also that the model structure can be subject- and time-dependent. This concerns the dimensionality of the state vector, the pattern of factor loadings, and in the pattern of interrelationships among latent states and latent residuals. As a consequence, the model-implied covariance matrix, and the model-implied mean vector are subject- and time-dependent. Theoretically, the model does thus accommodate full heterogeneity within and between subjects. We now impose increasingly restrictive invariance constraints relating to the dimensions time and subject. We first consider the model constraints that lead from total heterogeneity to MI over time and subjects. We then consider the additional model constraints that eventually result in full invariance over time and subjects, i.e., an ergodic process, as discussed by Molenaar and colleagues (e.g., Molenaar, 2004; Molenaar and Campbell, 2009). The different models are organized in form of a taxonomy. Figure 1 represents this taxonomy in terms of model equations and verbal terms. As we are interested in the conditions that establish equivalence between the intra- and inter-individual level of analysis, we focus on those models in which we impose constraints simultaneously within and between subjects.

### Modes of equivalence between the intra- and inter-individual level of analysis

We first consider the baseline model as a reference. As presented in Equations (2) and (3) neither the measurement model nor the latent process model is restricted over time or over subjects. Note that, technically, the model is not identified until some sort of time-related pattern is imposed. Assuming some pattern would also be indicated from a theoretical perspective. This needs however not involve constraining (measurement) model parameters to be time-invariant. There is thus no equivalence relationship between the intra- and the inter-individual level. A model based on pooled data over occasions and subjects would address a process that is a mixture over time and subjects unconditional and conditional on the latent process (cf. Muthén, 1989). Applying the interpretation of MI as unbiasedness results in the following conclusions. The absence of MI over time within subjects due to time-varying measurement parameters indicates that within any given person there is systematic observed variability over time that is not attributable to the targeted latent variables in **η***_i, t_*. Since MI over subjects within time points does also not hold due to person-specific measurement parameters there is systematic observed variability between persons that is not attributable to the targeted latent variables. Different time- and subject-varying variables may cause measurement variance and these associations may be person- and indicator-specific and may change over time. As long as these (unknown) variables and their effects on the indicators are not accounted for, the interpretation of the latent variables as they develop over time and differ over subjects remains complicated. This is in accordance with the notion of MI as theoretical equivalence which holds that the latent variables in **η***_i, t_* are not necessarily interpretable in an invariant sense over time or subjects. That would become directly apparent in an extreme case, in which the measurement model would display different factor loading patterns over time or subjects. In the discussion, we elaborate on recently suggested strategies to handle and explore such a situation.

By constraining all parameters to be invariant over time and subjects we obtain the extreme opposite. The measurement and process model reduce to

(4) $${\text{y}}_{i,\;t}=\tau +\Lambda \eta_{i,\;t}+\varepsilon_{i,\;t}$$

and

(5) $$\eta_{i,\;t}=\alpha +\text{B}\eta_{i,\;t-1}+\zeta_{i,\;t}$$

with

$$\begin{array}{l} \varepsilon_{i,\;t}\;\sim \;N\text{​}\left(0,\;\Theta \right),\\ \zeta_{i,\;t}\;\sim \;N\text{​}\left(0,\;\Psi \right). \end{array}$$

An additional requirement ensuring stationarity of the latent process, i.e. time-invariant process characteristics, is that all eigenvalues of matrix **B** are less than one in absolute value (Hamilton, 1994; Molenaar, 2004). Note that the model-implied distributions of observed and latent variables are now independent of subject and time. This model thus represents an operationalization an ergodic process under the assumption of normality (Molenaar, 2004, p. 208). Under these conditions one (intra-individual) process model generalizes across the entire time span and across all subjects in the population considered, i.e., the individual state-space time series models coincide with a standard between-subject longitudinal factor model based on at least two occasions (Molenaar et al., 2003; Molenaar, 2004). Consequently, the between-subject model provides a description of the intra-individual dynamics of each individual in the population and over the entire period of time considered (e.g., Molenaar, 2004; Hamaker et al., 2005; Molenaar and Campbell, 2009). Pooling over persons and time points is feasible as modeling results are fully generalizable between and within subjects.

Between these two extreme variants is the model in which the invariance constraints only concern the measurement model. Strict FI imposed simultaneously with respect to time and subject implies MI with respect to time and subject and results in the model

(6) $${\text{y}}_{i,\;t}=\tau +\Lambda \eta_{i,\;t}+\varepsilon_{i,\;t}$$

and

(7) $$\eta_{i,\;t}=\alpha_{i,\;t}+{\text{B}}_{i,\;t}\eta_{i,\;t-1}+\zeta_{i,\;t}$$

with

$$\begin{array}{l} \varepsilon_{i,\;t}\;\sim \;N\text{​}\left(0,\Theta \right),\\ \zeta_{i,\;t}\;\sim \;N\text{​}\left(0,\;\Psi_{i,\;t}\right). \end{array}$$

Note that the conditions for MI over time and subjects concern only the measurement process, that is, invariance of the model parameters over time and subjects conditional on the latent process. Simultaneous MI over time and subjects thus represents a form of structural equivalence between levels of analysis that still allows for substantial heterogeneity with respect to the latent variables and their interrelations over time and over subjects. Consequently, we propose to distinguish between two *modes* of structural equivalence. That is, a mode of measurement equivalence, which involves MI over time and subjects but does not include equivalence of the interrelations among the latent variables and latent residuals, and a distinct mode of full equivalence, which is ergodicity. A model based on data pooled over occasions or subjects would imply a latent process that is a mixture over time and subjects whereas modeling results regarding the measurement process would be generalizable over time and subjects.

Interpreting MI as biasedness of the indicators, this model implies that systematic observed intra-individual as well as inter-individual variability is attributable to the targeted latent variables in **η***_i, t_*. The interpretation as theoretical invariance holds that the same latent variables are measured within and between subjects. Systematic within- and between-subject variation can be viewed as variation on the same set of latent variables (cf. Lubke et al., 2003a). The model would thus capture intra-individual dynamics and inter-individual differences therein with respect to the targeted latent variables (cf. Hamaker et al., 2007). In this sense, measurement equivalence could be considered a necessary condition for studying intra- and inter-individual differences pertaining to the latent variables of interest.

## Illustration

### Purpose of illustration, data description, and selection

We show how measurement invariance can be investigated (a) over subjects and (b) over time within a given subject. As we use a modeling approach for stationary time series data we shall limit our illustration to time series models which we assume to be invariant with respect to time. We demonstrate below, that these models allow us to incorporate measurement variance over time to a limited extent.

We use data from Borkenau and Ostendorf (1998) that consist of individual time series of self-ratings on personality items. On 90 successive days, 22 students indicated the degree to which 30 adjectives applied to their daily state. Standard between-subject factor analysis showed that the items measure the inter-individual difference traits Neuroticism, Extraversion, Agreeableness, Conscientiousness and Openness to Experience (e.g., Borkenau and Ostendorf, 1990; McCrae and John, 1992; Borkenau and Ostendorf, 1998). The response format was a 7 point scale with high scores indicating high correspondence between described and perceived state.

For our present illustration, we consider a subset of items and subjects with approximately continuously and normally distributed responses, and the absence of obvious mean-level-trends or variability-changes in the series over time^4^. We focus on three individuals (subjects 7, 13, and 22), and their responses to the extraversion (“dynamic,” “sociable,” “shy,” “silent,” “lively,” “reserved”) and agreeableness marker items (“selfish,” “good-natured,” “domineering,” “helpful,” “obstinate,” “considerate”). The individual data and descriptive figures are available as supplementary materials.

### Determining the individual state-space time series models

To set up the individual models, we imposed a two-factor measurement model on each individual's data, such that the extraversion marker items load on one, the agreeableness marker items on a second factor. Note that there is no guarantee that the two-factor model, which would be expected to fit the data in standard inter-individual factor analysis, will fit the individual time series data (e.g., Molenaar, 2004; Hamaker et al., 2005; Molenaar and Campbell, 2009). By means of exploratory factor analysis, one could identify individual factor solutions that would potentially be person-specific (regarding sets of factors and factor loading patterns) and then conduct within-person fit comparisons between the individual models and the two-factor model (e.g., Hamaker et al., 2005, 2007). Here, we assume configural invariance over individuals, that is, an invariant number of factors and an invariant factor loading pattern (Meredith, 1993).

**Figure 2:**
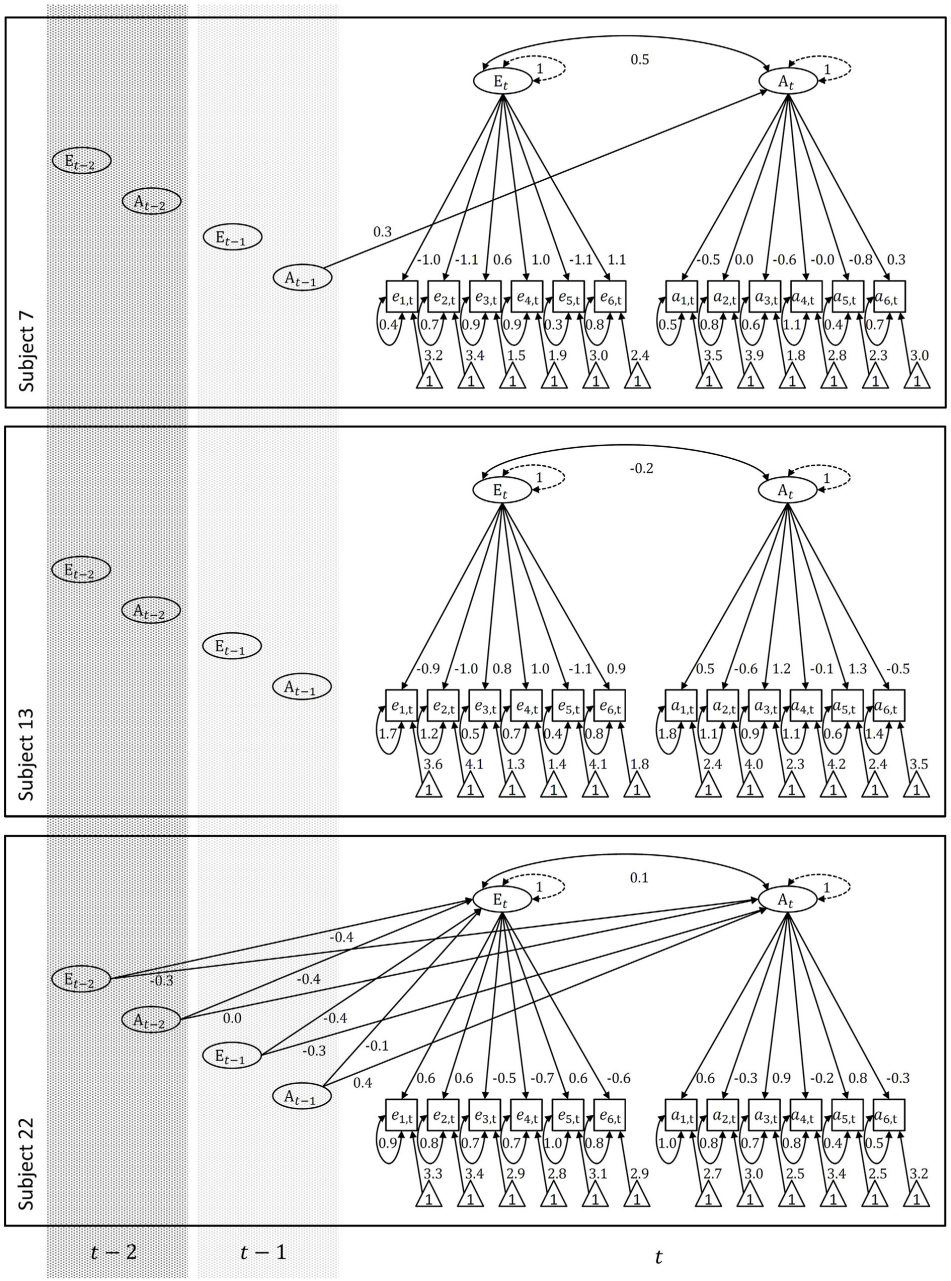
**Relatively best fitting models for subjects 7, 13, and 22**. Paths fixed to zero are not drawn. Note that these include the regression parameters of the vector eta on the constant, i.e., vector alpha, which are fixed to zero for scaling purposes. Paths fixed to one are dashed. These include the latent residual variances in order to provide a latent metric. Freely estimated paths are drawn in black and parameter point estimates are provided. Items denoted with e are extraversion marker items, whereas items denoted with a are agreeableness marker items. The numerical ordering of the items employed here corresponds to the ordering of the items as given in the data description section. Index *i* is dropped as the models describe single individuals.

We determined the individual process models by modeling the auto- and cross-lagged relationships among the factors using the Fortran program MKF (Dolan, 2010)^5^. This program can fit linear stochastic time series models in state-space format to stationary time series data via the linear, time-invariant Kalman filter algorithm. For correctly specified state-space models the Kalman filter provides optimal estimates of the latent variable states over time and gives rise to ML estimates of the model parameters. Detailed explanations of the estimation procedure can for instance be found in the econometric (e.g., Harvey, 1989; Hamilton, 1994; Durbin and Koopman, 2001) and psychometric literature (e.g., Oud et al., 1990; Chow et al., 2010). Within each individual we contrasted vector auto-regressive processes of first order (VAR(1)), second order (VAR(2)), and of order zero (VAR(0)). In the last case, the factors do not display lagged relationships. We pruned models by fixing to zero non-significant relationships in **B***_i_* and **Ψ***_i_* (overall-α = 0.05). We imposed scaling by fixing the latent intercepts to zero and the latent residual variances to one. The information criteria BIC (Schwarz, 1978) and AIC (Akaike, 1974) served as main indicators for relative model fit but we also conducted Log-Likelihood difference tests where models were nested (α = 0.05). Table 1 provides an overview of the results and Figure 2 shows path diagrammatic representations of the individual models.

**Table 1:** **Comparison of different process models within individuals**.

**Process model**	**npars**	**−2LogL**	**AIC**	**BIC**	**χ^2^-increase (relative to)**	***df***	***p***
**Subject 7**							
VAR (0)	37	1089	1163	1255	10.377 (VAR (1))	4	0.035
					6.993 (VAR (1)*)	1	0.008
VAR (1)	41	1079	1161	1263			
VAR (1)*	38	1082	1158	1253	3.384 (VAR (1))	3	0.336
VAR (2)	45	1095	1185	1297			
**Subject 13**							
*VAR (0)*	37	1522	1596	1689	5.221 (VAR (1))	4	0.265
VAR (1)	41	1517	1599	1702			
VAR (2)	45	1515	1605	1718			
**Subject 22**							
VAR (0)	37	1212	1286	1378	23.655 (VAR (1))	4	0.000
VAR (0)*	36	1214	1286	1376	1.815 (VAR (0))	1	0.178
VAR (1)	41	1188	1270	1373			
VAR (1)*	37	1202	1276	1368	13.366 (VAR (1))	4	0.010
*VAR (2)*	45	1161	1251	1363.7			
VAR (2)*	39	1189	1267	1364.1	27.390 (VAR (2))	6	0.000

*Model variants denoted with an asterisk are pruned with respect to simultaneous and lagged relationships. The relatively best fitting model according to AIC and BIC is set in italics. χ ^*2*^-differences are reported for nested models*.

According to AIC and BIC, subjects 7 and 22 both display a latent process that involves lagged relationships among the factors. For subject 7 there is only one auto-regressive effect of first order for the agreeableness factor, for subject 22 there is the full set of first- and second-order auto- and cross-lagged regression effects. In case of subject 13 the latent process does not contain any lagged effects among the factors. Within occasions, both factors are correlated within each of the three subjects.

With respect to the individual measurement models, the loadings relating the extraversion indicators to the corresponding factor seem to be relatively homogeneous and reasonably large within each individual (although the measurement residual variances are consistently large). This is different for the agreeableness indicators which are associated not only with more heterogeneous loadings but also with loadings close to zero as in case of the item “helpful.” Especially for subject 7 it is questionable whether one coherent dimension underlies his or her responses to the agreeableness indicators. However, to test this we would have to employ a more explorative approach as outlined above. Note that the loading signs suggest that the factors are inverted in some cases.

### Addressing MI over subjects

To address MI over subjects we made use of the multi-group modus in MKF treating each individual as a group. FI was then tested via pairwise comparisons between all three subjects. Since we scaled in the latent space by standardizing the conditional latent states, all factor loadings and measurement intercepts are freely estimated and can thus all be subjected to a test of invariance across groups (Raykov et al., 2012). In order to not confound FI constraints with invariance constraints pertaining to the latent level, we freely estimated the latent residual variances in one of the subjects whenever the factor loadings were constrained to equality. Equivalently, we freed the latent intercepts in one of the models, whenever the measurement intercepts were constrained to equality (Wicherts and Dolan, 2010; Raykov et al., 2012). Table 2 provides an overview of the results.

**Table 2:** **Multi-group models with measurement parameters constrained over groups**.

**Measurement models**	**npars**	**−2LogL**	**AIC**	**BIC**	**χ^2^-increase (relative to)**	***df***	***p***
**Comparison between subjects 7 and 13**							
Configural invariance	75	2604	2754	2942			
*Weak FI* (**Λ***invariant*)	65	2621	2751	2913			
Strong FI (**Λ, τ** invariant)	55	2797	2907	3044			
Strict FI (**Λ, τ, Θ** invariant)	43	2863	2949	3056	66.087(Strong FI)	12	0.000
**Comparison between subjects 7 and 22**							
Configural invariance	83	2242	2408	2616			
*Weak FI* (**Λ***invariant*)	73	2255	2401	2583			
Strong FI (**Λ, τ** invariant)	63	2474	2600	2757			
Strict FI (**Λ, τ, Θ** invariant)	51	2516	2618	2745	42.156(Strong FI)	12	0.000
**Comparison between subjects 13 and 22**							
Configural invariance	82	2684	2848	3053			
*Weak FI* (**Λ***invariant)*	72	2701	2845	3025			
Strong FI (**Λ, τ** invariant)	62	2787	2911	3066			
Strict FI (**Λ, τ, Θ** invariant)	50	6162	6262	6387	3374.630(Strong FI)	12	0.000

*The relatively best fitting model according to AIC and BIC is set in italics. χ *^2^*-differences are reported for nested models*.

For all pairwise comparisons between subjects, the AIC and the BIC favored the weakly factorial invariant model. Note that a **χ**^2^-difference-test for instance between the configurally invariant and the strictly factorial invariant model cannot be conducted as the models are not nested. This is due to the freely estimated latent parameters in the strictly factorial invariant model (Raykov et al., 2012). The finding of subject-invariant factor loadings suggests that the same dimensions underlie the variation within each of the three individuals (Hamaker et al., 2007). These are however not necessarily the dimensions underlying the differences between individuals (Lubke et al., 2003a; Hamaker, 2007) as, according to the fit indices used, uniform bias is likely to be present for at least some of the items. Meaningful comparisons between subjects can be considered feasible as long as they refer to differences in the structure of latent intra-individual variation only. The extent and nature of potential uniform bias between individuals could be the subject of subsequent analyses.

### Addressing MI over time

Strict FI over occasions cannot be tested directly, as we confined this illustration to time-invariant models. However, we can investigate whether strict FI over time is violated in a specific sense. We do this by testing for uniform bias of the indicators with respect to a selected time-varying variable *X*. This can be cast in terms of a main-effect of *X* on the indicators additionally to the latent variables (Lubke et al., 2003b).

We extend the time-invariant model for a given individual *i* = i^*^ to

(8) $${\text{y}}_{i^{*},\;t}=\tau_{i^{*}}+\Lambda_{i^{*}}\eta_{i*,\;t}+\Gamma_{i*}x_{i^{*},\;t}+\varepsilon_{i^{*},\;t}$$

and

(9) $$\eta_{i^{*},\;t}=\alpha_{i^{*}}+{\text{B}}_{i^{*}}\eta_{i^{*},\;t-1}+\Phi_{i^{*}}{\text{x}}_{i^{*},\;t}+\zeta_{i^{*},\;t}$$

where *x*_i*, *t*_ is a *r* × 1 vector of (fixed) covariates and **Γ**_i*_ and **Φ**_i*_ are *p* × *r* and *q* × *r* matrices of regression coefficients. If there is a significant effect of at least one variable in *x*_i*, *t*_ on at least one of the indicators, measurement invariance over time would be violated, as—returning to Mellenbergh's definition—the distribution of the indicators is dependent on *x*_i*, *t*_ conditional on the latent variables (Lubke et al., 2003b). However, the absence of uniform bias with respect to *x*_i*, *t*_ implies neither MI with respect to these variables (which may still introduce non-uniform bias or be associated with varying measurement residual variances), nor MI with respect to other time-varying variables, let alone MI with respect to time.

**Figure 3:**
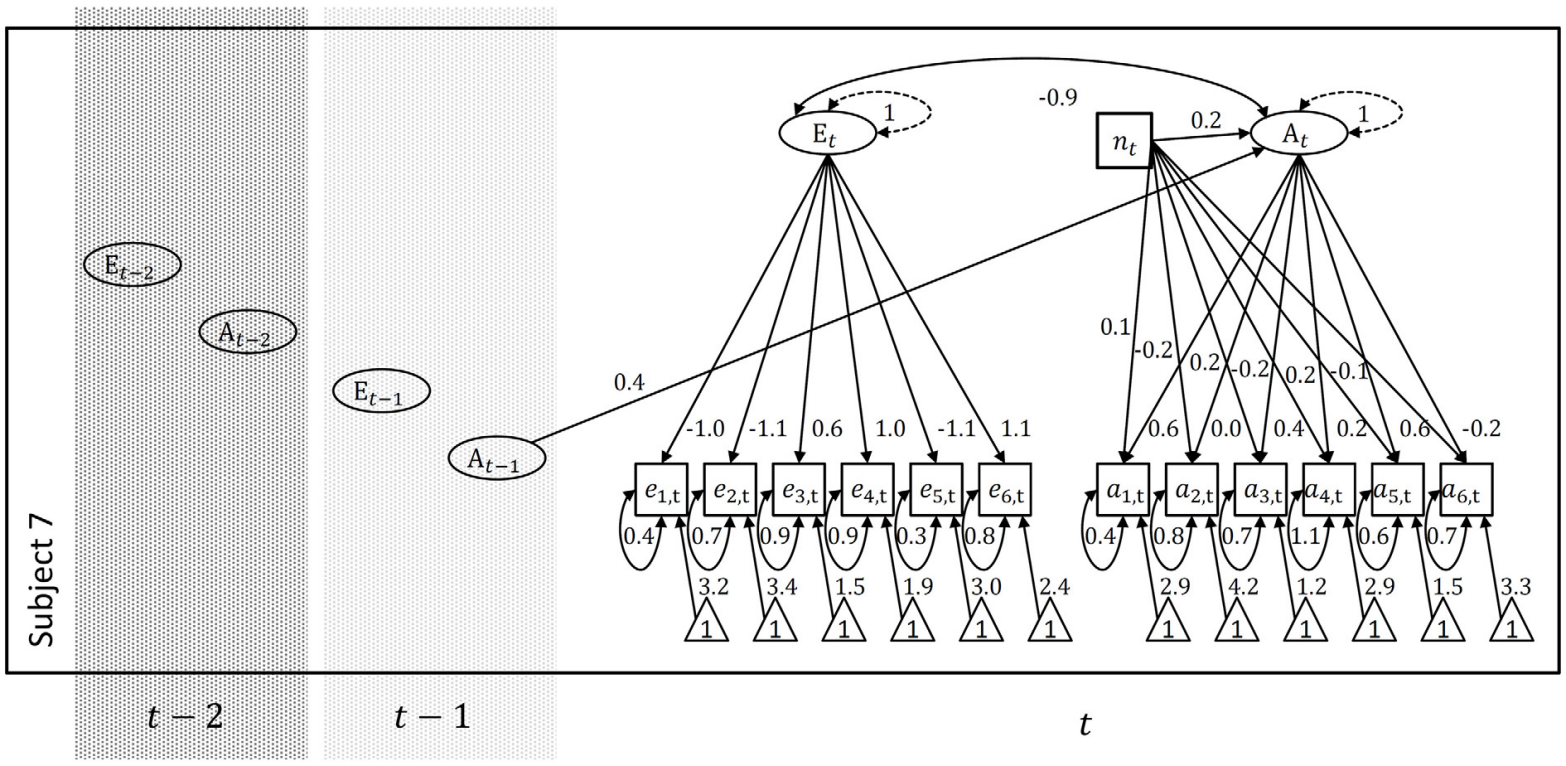
**Individual model for subject 7 including the neuroticism marker item “bad tempered” as a potentially biasing (fixed) variable**. According to this representation, the neuroticism item possibly affects the agreeableness marker items above the potential effect it has through the agreeableness factor.

We focused on the neuroticism marker item “bad tempered” as a mood indicator and potentially biasing variable in subject 7. The results are shown in Table 3 and the path diagrammatic representation of the corresponding model is displayed in Figure 3.

**Table 3:** **Comparison of models incorporating a potentially biasing variable x for subject 7**.

**Model**	**npars**	**−2LogL**	**AIC**	**BIC**	χ**^2^-increase (relative to)**	***df***	***p***
*y*, **η** on *x*	52	1010	1114	1244			
**η** on *x*	40	1044	1124	1224	34.250 (*y*, **η** on *x*)	12	0.001
*y*(*a*), **η**(*a*) on *x*	45	1034	1124	1237			
**η**(*a*) on ***x***	39	1049	1127	1225	15.061 (***y***(*a*), **η**(*a*) on *x*)	6	0.020

**y*(a) denotes the agreeableness marker items, and **η**(*a*) denotes the agreeableness factor. We allowed for direct effects of *x* on the latent variables but did not establish whether these were significant*.

***χ**^2^-differences are reported for nested models*.

The BIC which is more responsive to parsimony than the AIC (Hamaker et al., 2005) favors the model without direct effect of the mood indicator on all indicators and the agreeableness indicators respectively. Both AIC and **χ**^2^-difference test suggest that uniform bias is present for at least one of the indicators. In a given modeling application one could investigate whether uniform bias can be accounted or controlled for also with respect to other potentially biasing covariates. Ultimately however, one needs to decide whether one is willing to discard other forms of bias over time as unlikely or whether actually a modeling approach that incorporates time-varying parameters is the more valid and more interesting alternative. Fitting the “wrong” model to intra-individual data which could be a measurement-invariant or more generally a time-invariant model, will also affect the quality of between-person comparisons. We briefly outline modeling approaches to time-varying dynamics in the discussion.

## Discussion

In this paper, we showed how MI (e.g., Mellenbergh, 1989), if present, may facilitate or, if absent, may complicate the generalizability of modeling results within and between subjects. Tying into the ergodicity debate (e.g., Molenaar, 2004), we clarified the relationship between the concepts of MI and ergodicity in the context of general latent variable modeling as well as in a linear multi-subject state-space time series model. We concluded that MI holding simultaneously over time and subjects implies a mode of structural equivalence between the intra- and the inter-individual level of analysis that is distinct from full structural equivalence, i.e., ergodicity. That is, measurement equivalence is a mode of structural equivalence conditional on the latent process. Following common interpretations of measurement invariance, the mode of measurement equivalence could be considered an important condition for integrative latent variable modeling of intra- and inter-individual differences (cf. Ellis and van den Wollenberg, 1993, who stress the importance of local homogeneity in IRT-modeling which is tantamount to measurement equivalence; cf. Millsap, 2011). Using intra-individual time series data from three individuals on daily personality states, we investigated the tenability of MI constraints over subjects and over time. Although strict FI over subjects was absent, the presence of weak FI suggested that between-subject comparisons were feasible with respect to the structure of latent intra-individual variation. We were limited in investigating MI over time due to the time-invariant models we employed. Consequently, we could test for specific MI violations but we did not address unbiasedness with respect to time.

The results of our illustration are in line with a growing body of empirical work investigating potential relationships between the structures of intra- and inter-individual variation and means. So, although we presented measurement equivalence as a less restrictive mode of equivalence between levels of analysis than full structural equivalence, we acknowledge that even this weaker form of structural equivalence may be overly restrictive. We can therefore only stress that the problem of non-ergodicity must in part be viewed as a measurement problem since the violation of measurement invariance with respect to time and subject is a source of heterogeneity within and between individuals (cf. Nesselroade et al., 2007, 2009; Borsboom et al., 2009). It was the aim of this paper to show that the investigation of measurement related heterogeneity within and between individuals in latent variable modeling qualifies as a problem which is related to but also distinct from the problem of ergodicity.

Regarding a closer examination of measurement related heterogeneity, the presented taxonomy is clearly an abstraction. In practice, the finding of untenable MI constraints is not necessarily the end of an investigation. Modeling application situations falling in the baseline model category and associated problems of measurement variance can be of very different nature. For instance, it may be possible to interpret measurement variance substantively against a given theoretical background (Millsap and Hartog, 1988; Kelderman and Molenaar, 2007). As an example, consider developmental or interventional effects over time, which may manifest as quantitative changes in given parameters, and, more importantly, in changes in the nature or meaning of the psychological entities of interest (Millsap and Hartog, 1988; Molenaar, 2004; Kelderman and Molenaar, 2007; Schmiedek et al., 2009). Also, even if measurement variance is considered a nuisance factor, only a few indicators may display measurement variance. Subsequent analyses may then locate the MI violation in the model and establish whether the number of unbiased indicators is sufficient to proceed with meaningful latent variable modeling, as we have indicated in the illustration (Byrne et al., 1989; Wicherts and Dolan, 2010). Likewise, not all subjects within a sample and not all occasions within a period of time may be affected by measurement variance. It may then be possible to identify intra- or inter-individual variables that explain measurement variance (Mellenbergh, 1989). In the present context, this relates to the concept of conditional equivalence introduced by Voelkle et al. (2014). In a simulation study these authors show that full equivalence between inter- and intra-individual model structures can easily be obscured by incorporating single factors that introduce subject- and time-related heterogeneity, e.g., linear mean trends over time, differences between groups of individuals. Conversely, it might be possible to identify such factors for certain constructs and control for them in order to establish conditional equivalence, that is, equivalence for subgroups of individuals and occasions. In case equivalence is well hidden or absent, one can still explore the various types of less restrictive (unconditional) relationships that may arise between intra-individual and inter-individual model characteristics (cf. Kuppens et al., 2010; Montpetit et al., 2010; Brose et al., 2014).

These approaches to the links between levels of analysis have yet to be utilized to specifically address measurement variance within and between individuals. To further emphasize why these approaches could be both interesting and necessary given measurement related heterogeneity within and between individuals, let us return to the assumptions, upon which MI is predicated. These concern the existence of the latent variables of interest and the appropriateness of the observed variables as indicators. The first premise holds, that the indicators are—although possibly imperfect, i.e., biased—valid in principle (cf. Meredith, 1964, 1993). That is, the indicators are to some extent measuring the variable they were designed to measure (Millsap, 2011) and these psychometric qualities should hold absolutely true or at least hold true for the units of analysis we wish to compare, say, a sample of individuals (Nesselroade et al., 2009). This in turn requires the assumption that the targeted latent variable is indeed given (Mellenbergh, 1989) or a theoretically sensible construct across the selected individuals. As noted by Byrne and Campbell (1999) these premises may be questionable, for instance in applying a measurement instrument in a setting, other than the setting in which it was developed. The setting may be determined by the cultural background of the examinees or the dimension of analysis, e.g., the intra-individual dimension. Hence, a violation of MI with respect to differing setting conditions can be indicative in the following regard. First, it may be that the given test is not valid under some conditions although the latent variable is—on an abstract level—existent or theoretically sensible. The latent variable simply manifests differently under different conditions (e.g., Byrne and Campbell, 1999). Nesselroade et al. (2007, 2009) pointed out that a targeted construct (e.g., athletic performance) may be a sensible choice for comparing different individuals—but may require the use of individual-specific indicators (“How well do you play tennis vs. golf?”). Second, a given test may be invalid under certain conditions because the construct is not conceptually sensible across conditions. To label these two scenarios, Byrne and Campbell (1999) refer to the term *construct bias* as opposed to item bias which indicates that the problem has shifted from an “operational” to a “theoretical” problem (Kelderman and Molenaar, 2007, p. 451). The concept of construct bias seems to be highly interesting when contrasting intra- and inter-individual variation. In the light of increasing empirical evidence in favor of substantive individual specifics (e.g., Hamaker et al., 2005; Brose et al., 2010) it raises the following question: To what extent are traditional psychological constructs (and according measurement instruments) that were derived in a between-subject context applicable to intra-individual differences? This is arguably a philosophical question, which has been addressed intensively by Borsboom et al. (2003, 2009) and by Cervone (2004, 2005). These authors argue that between-subject constructs like extraversion and agreeableness do well in describing inter-individual differences, but are problematic at the level of the individual, where they lack “causal force” (e.g., Cervone, 2004; p. 184). That is, *per se*, they do not map onto specific psychological mechanisms or processes within the individual, and are thus not suitable to feature as explaining factors in a within-subject model of psychological functioning (van der Maas et al., 2006; Borsboom et al., 2009). Borsboom et al. (2009) conjecture that there are “infinitely many ways” (p. 88) to achieve a certain outcome on a standard between-subject dimension. The associated constructs thus may lack coherence from an individual-driven perspective, in that they emerge as abstract aggregates only at the level of the population. However, this pessimistic prospect regarding the meaningful application of inter-individual level constructs to the individual can be probed empirically. Millsap employs the term *differential item functioning* rather than the term bias to indicate that “the researcher is unable or unwilling to clearly define the targeted attribute” (Millsap, 2011; p. 9). This can be turned into a positive message, namely to explore measurement variance—be it within or between individuals—as a potentially meaningful phenomenon.

An explorative empirical approach to person- and time-related heterogeneity at the level of measurement using the above described strategies and principles can enlighten how measurement instruments that were constructed in the between-subject context function at the within-subject level. This in turn can inform (and be informed by) the elaboration of individual-level concepts and theories (e.g., Cervone, 2005) as well as their implementation in empirical research in terms of operationalizations, measurement devices, and modeling techniques (e.g., Schmiedek et al., 2009). In this sense, it could contribute to building up the theoretical and conceptual foundation that is needed for a true reorientation toward the individual in differential psychology (Molenaar, 2004).

The presented modeling approach has the following limitations, however, that would restrict such an explorative endeavor. First, we based our modeling on the linear, time-invariant Kalman filter and ML estimation which led to time-invariant time series models. Time-varying model parameters can—to some extent—be accommodated using the extended Kalman filter (e.g., Chow et al., 2011; Chow and Zhang, 2013) or a Bayesian approach (e.g., Del Negro and Otrok, 2008). Second, we employed a multi-group approach, i.e., a two-step procedure to address inter-individual differences in intra-individual dynamics. Inter-individual differences in intra-individual model parameters can be quantified and modeled directly using a Bayesian multi-level approach (e.g., Lodewyckx et al., 2011). Note, however, that multi-group modeling is in principle less restrictive than hierarchical modeling. In the present context, it did not impose any restrictions across individuals apart from applying the same modeling framework to each individual's data. That is, within individuals, we assumed continuous, normal variables, at the manifest and latent level, which were linearly related to each other. Our reliance on the linear factor model here is expedient, although we are satisfied linear modeling of 7 point scales is adequate. Generalized linear modeling of intra-individual time series to accommodate discrete indicators is possible (cf. van Rijn et al., 2010), but at present depends on software development. Non-normally distributed continuous indicators (due to nonlinear effects) can be approximated by mixtures of (un-)conditional normal distributions (e.g., Klein and Moosbrugger, 2000). Note that in our case of single-subject models, mixture models return us to time-varying models (Hunter, 2014), which are increasingly discussed in the psychometric literature.

### Conflict of interest statement

The Review Editor Ellen Hamaker declares that, despite having been supervisor of author Noémi K. Schuurman, who is also affiliated with the same institution and whom they collaborated with, the review process was handled objectively and no conflict of interest exists. The authors declare that the research was conducted in the absence of any commercial or financial relationships that could be construed as a potential conflict of interest.

## Footnotes

^1^This useful notion was suggested by one of the reviewers.

^2^The shift in perspective from MI with respect to specific variables to MI over subjects or time has interesting implications (cf. Meredith, 1993, p. 529, theorem 3). MI over subjects implies MI with respect to any variable that varies exhaustively over subjects within the population considered. Equivalently, and under the assumption of an appropriate sampling rate over time, MI over time implies MI with respect to any variable that varies exhaustively within the period of time considered. Hence, by taking this perspective, one automatically accounts for all measured or unmeasured (discrete and finite) background variables that vary along the dimensions time and subject (cf. Lubke et al., 2003b).

^3^Under the assumptions that multivariate normality holds, it is unlikely that variation in measurement error variance and variation in specific factor variance cancel each other out across occasions and subjects respectively, and it is unlikely that variation in measurement intercepts and variation in specific factor means cancel each other out across occasions and subjects respectively (cf. Meredith, 1993; Lubke et al., 2003a,b).

^4^We selected subjects based on visual inspection of the frequency distributions and time series plots of their responses. Although the five factor marker items may be considered discrete, they are often treated as continuous in the literature (e.g., Borkenau and Ostendorf, 1998; Hamaker et al., 2005, 2007; Rammstedt and John, 2005). Indeed, Dolan (1994) demonstrated, that treating indicators with at least seven ordered response categories as continuous, does not affect standard errors and overall test statistics of normal theory maximum likelihood estimation—if the distribution of each indicator is not too skewed. Lubke and Muthén (2004) investigated problematic effects of skewed indicator distributions of pseudo-continuous items in standard confirmatory factor analysis.

^5^The program (including documentation) is available by request from c.v.dolan@vu.nl. All MKF in- and output files for the models fitted are available as supplementary materials. These also include R-code to set up data and input files for MKF, execute MKF, and read MKF output files.
