# Supplementary material for: Measurement invariance within and between individuals: a distinct problem in testing the equivalence of intra- and inter-individual model structures
Source: Front Psychol. 2014 Sep 19;5:883. doi: 10.3389/fpsyg.2014.00883 (PMC4193237; doi:10.3389/fpsyg.2014.00883)
Supplement: Supplementary file 1 [file DataSheet1.ZIP › MIadolf_supplements.docx]

***Supplementary Material***

**Measurement invariance within and between individuals:
A distinct problem in testing the equivalence of intra- and inter-individual model structures.**

**Janne Adolf^1^*, Noémi Schuurman^2^, Peter Borkenau^3^, Denny Borsboom^4^ and Conor Dolan^5^**

^1^Center for Lifespan Psychology, Max Planck Institute for Human Development, Berlin, Germany
^2^Department of Methodology and Statistics, Faculty of Social and Behavioral Sciences, Utrecht University, Utrecht, The Netherlands
^3^Personality and Diagnostics Group, Department of Psychology, Faculty of Philosophy I, Martin-Luther-University Halle-Wittenberg, Halle, Germany
^4^Psychological Methods Group, Department of Psychology, Faculty of Social and Behavioral Sciences, University of Amsterdam, Amsterdam, The Netherlands
^5^Department of Biological Psychology, Faculty of Psychology and Education, Free University of Amsterdam, Amsterdam, The Netherlands

*** Correspondence**: Janne Adolf, Center for Lifespan Psychology, Max Planck Institute for Human Development, Lentzeallee 94, 14195 Berlin, Germany.
adolf@mpib-berlin.mpg.de

Highlights

The following points summarize the specific contribution of our paper to the special issue on measurement invariance

- We discuss how the concept of measurement invariance can generally and operationally be applied at the intra-individual and the inter-individual level of analysis.
- We show that measurement invariance holding simultaneously over time and subject can be interpreted as constituting a mode of structural equivalence between the intra- and the inter-individual level of analysis that is distinct from full structural equivalence.
- We illustrate how measurement invariance can be tested over subjects and over time in the context of (time-invariant) multi-subject time series models in state-space format.
- We strengthen the view that heterogeneity between and within subjects is a measurement problem that can be addressed in the context of latent variable modeling.

**Supplementary figures**

The following supplementary figures show the raw time series and frequency distributions of all individual’s responses over time.

**Supplementary figure 1.** Responses to extraversion marker items as provided by subject 7.

**
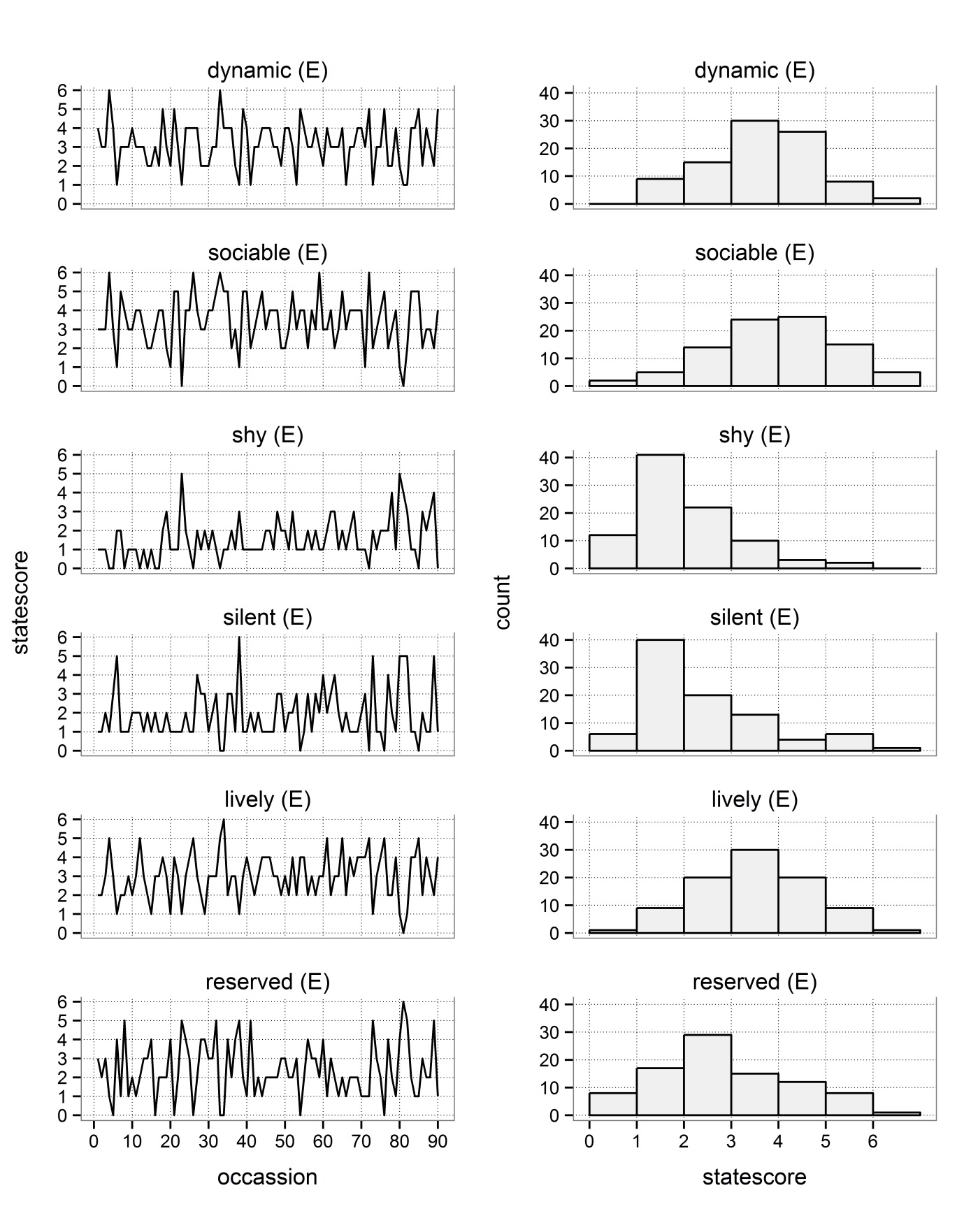
**

**Supplementary figure 2.** Responses to agreeableness marker items as provided by subject 7.


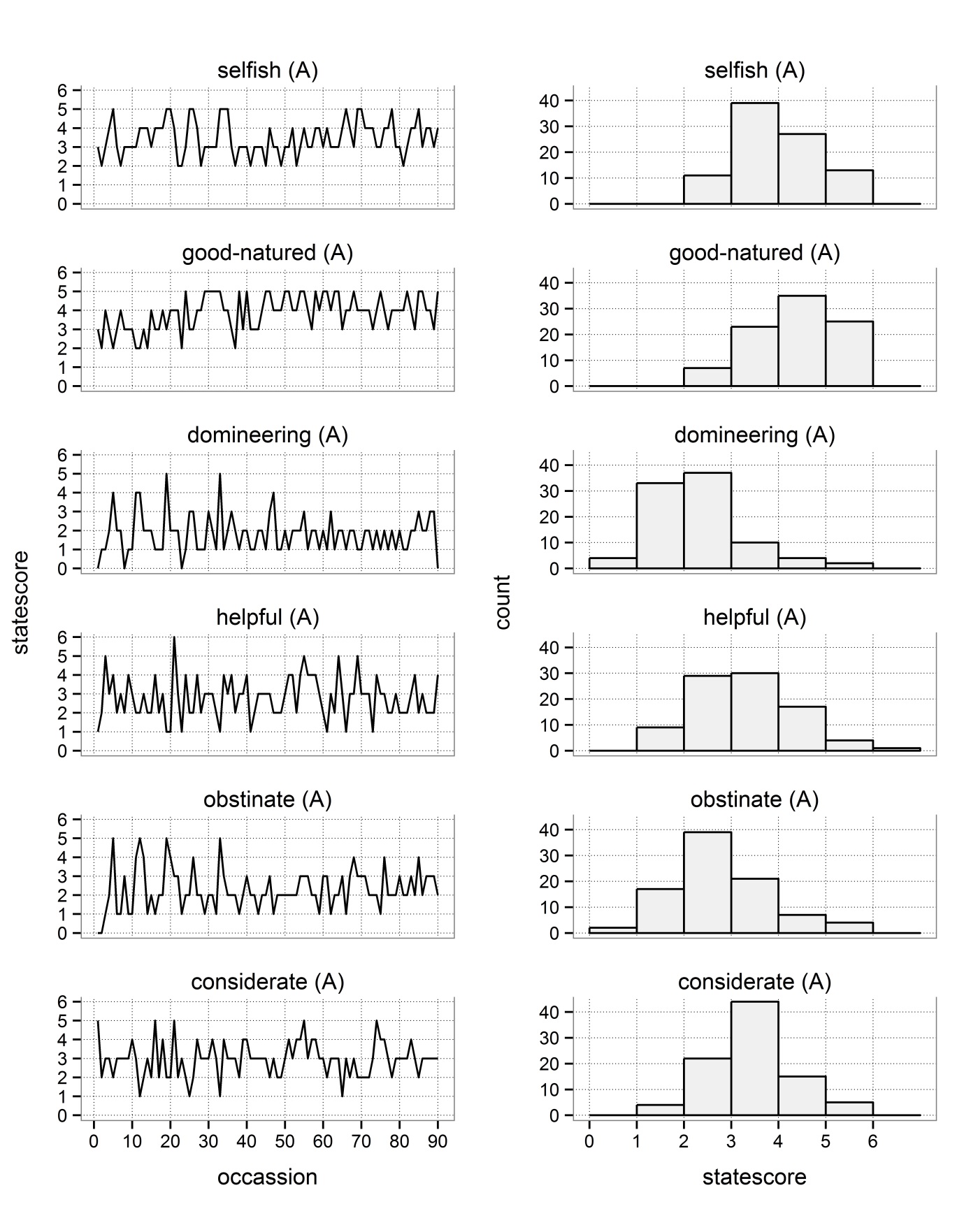


**Supplementary figure 3.** Responses to extraversion marker items as provided by subject 13.


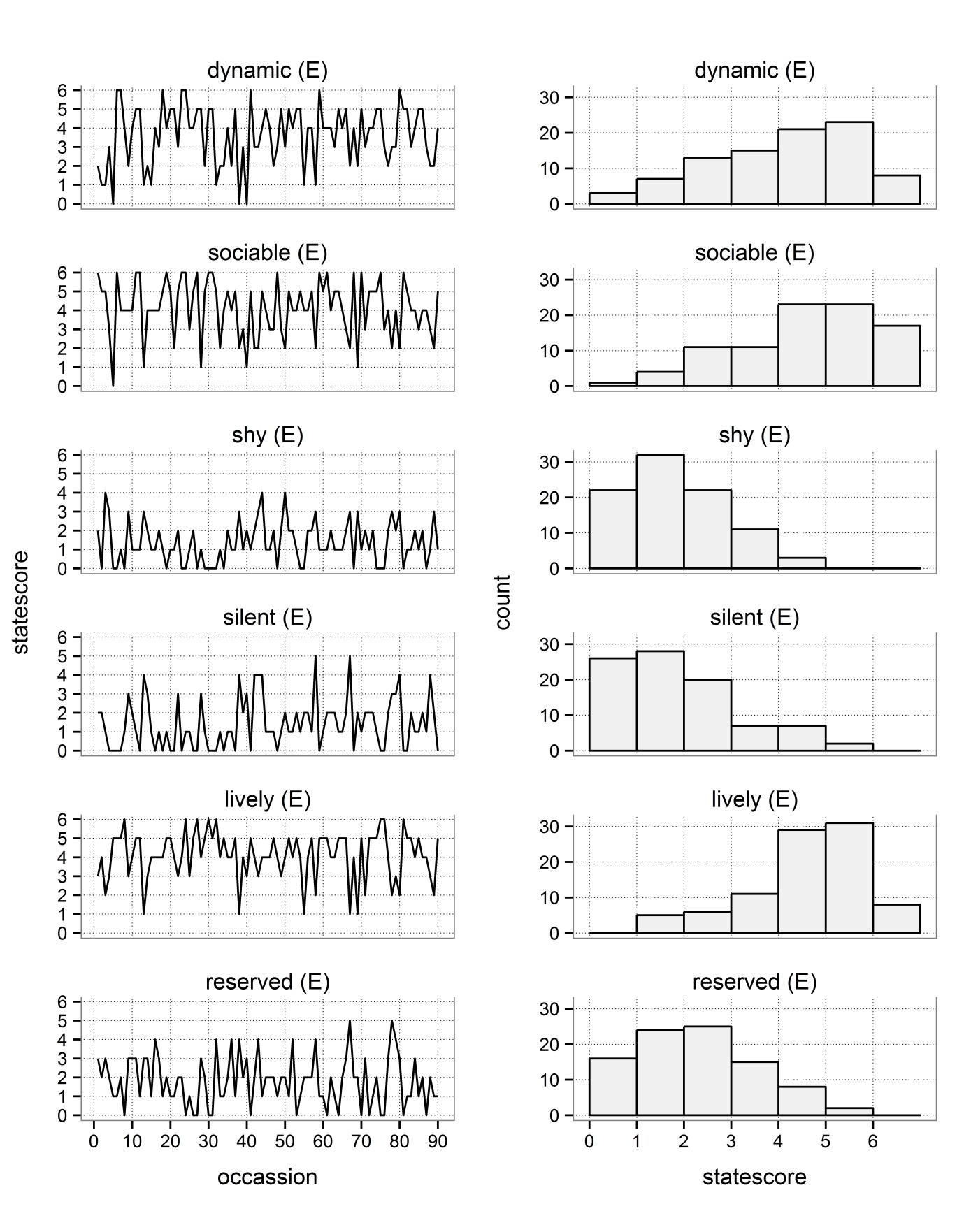


**Supplementary figure 4.** Responses to agreeableness marker items as provided by subject 13.


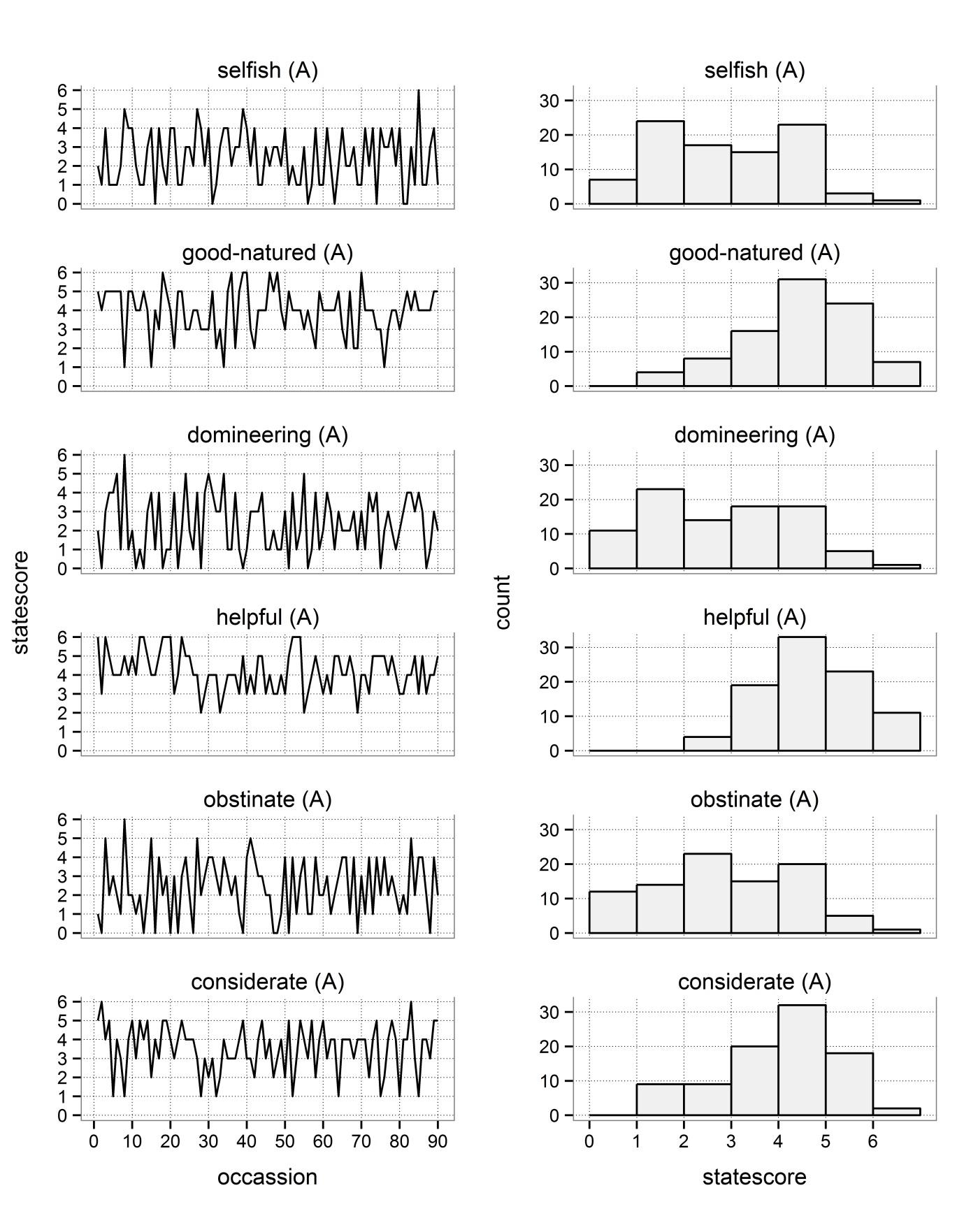


**Supplementary figure 5.** Responses to extraversion marker items as provided by subject 22.


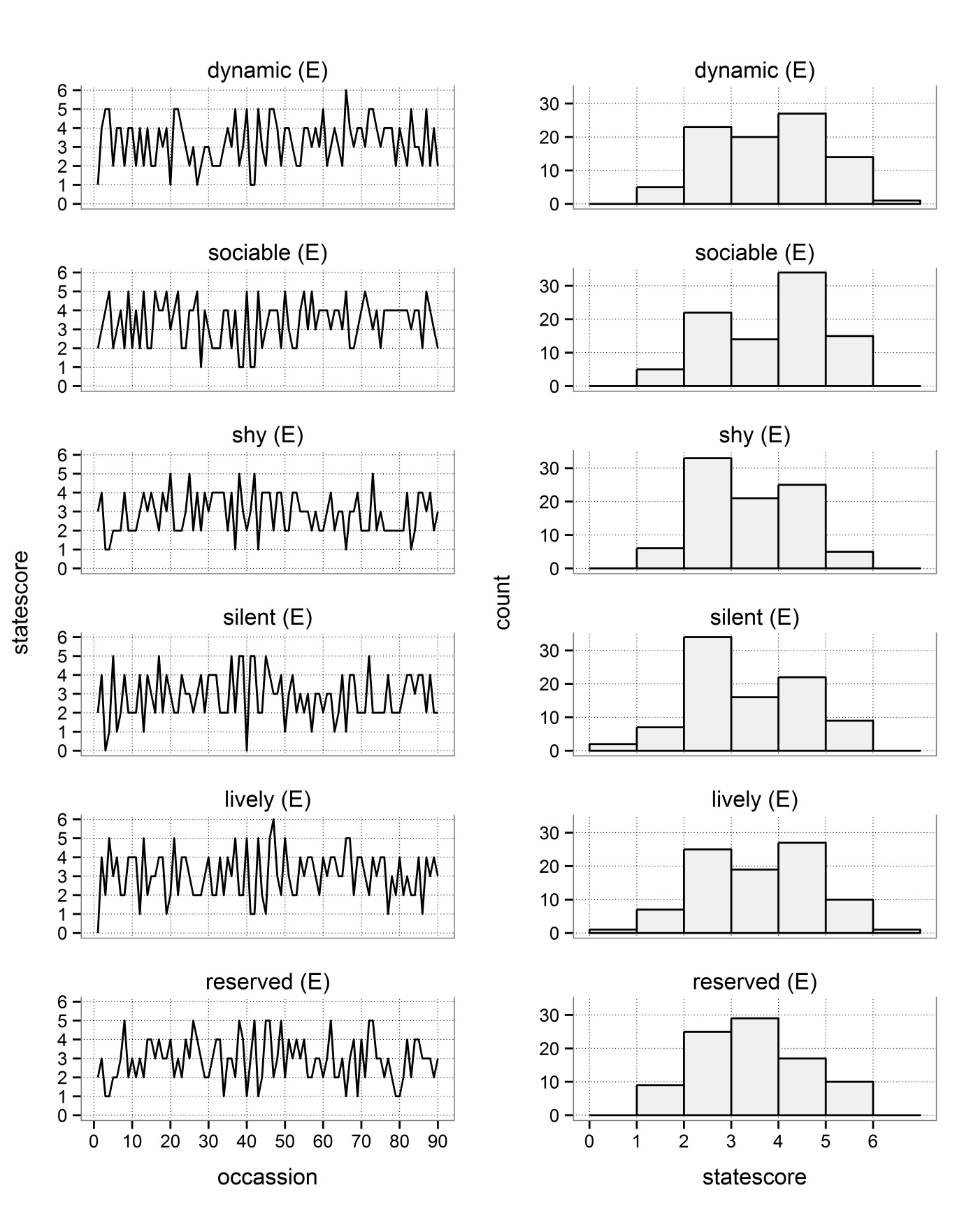


**Supplementary figure 6.** Responses to agreeableness marker items as provided by subject 22.


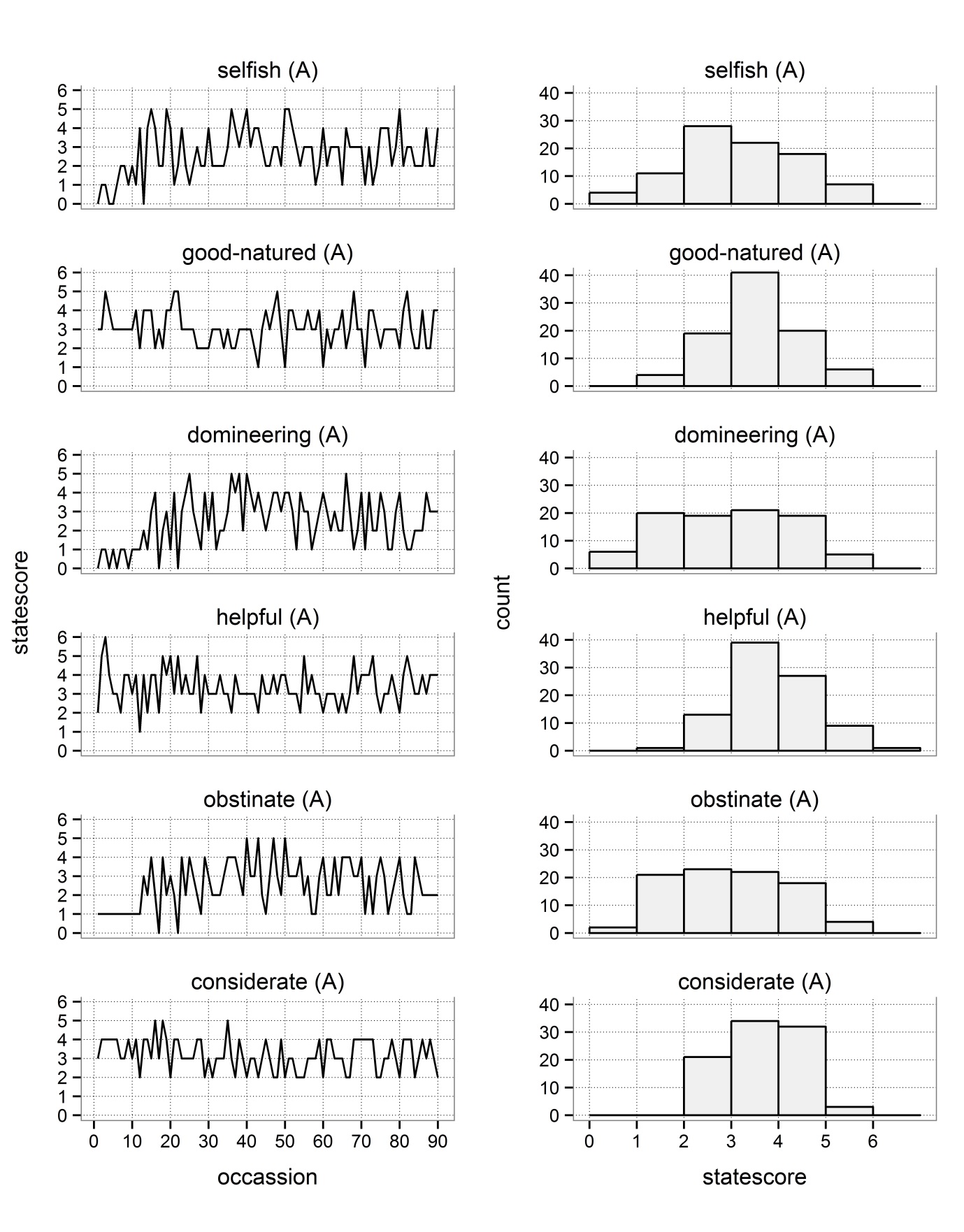


**Data**

Data files in MKF-format available for subjects 7, 13, and 22 and selected items.

**MKF via R**

The following R-code can be used to set up data and input files for MKF from R, execute MKF under R, and read MKF output files into R (also available file MKFviaR.R).

#*********************************#

# MKFM6-1-WRAPPER-LIKE-CODE FOR R #

#*********************************#

# INFORMATION TO BE SPECIFIED

#------------------------------------------------------------------------------

path1<-"C:/User/folderA" # working directory;

# must contain "mkfm6-1.exe", datafiles (in suitable format, see manual), input-files (in suitable format, see manual or use this script)

path2<-"C:/User/folderB" # directory to save the results-table in

nt=100 # number of time points / length of time series

ny=6 # number of manifest variables

ne=2 # number of latent factors

nx=0 # number of time-varying covariates

name.inf="inp1" # name of mkfm-inputfile; input-files can be created using this code (otherwise to be prepared manually, see manual)

name.ouf="oup1" # name of mkfm-outputfile; will be created by this code)

name.df="dp1" # name of data file

# IF you have a data-object in R and want to write data-files for mkfm

write.data=0 # writes data-files if and only if write=1

data_mkf<-data.p1 # R-object containing the data in in long-format (observations in rows, variables in columns)

cvec=matrix(0,ne,1) # vector containing starting values for the latent variables (expected values at t0) ! WRAPPER NEEDS TO BE EXTENDED FOR AR(>1)

# IF you want to set-up the input-files from R; DEFAULTS: missings are expected to be -999, se=yes, rf=yes, it=1000 (see manual)

write.input=0 # sets up input-files if and only if write=1

ngroup=1 # number of groups ! WRAPPER NEEDS ADJUSTMENT FOR NGROUP>1

nm=1 # number of model/index of group model

ns=1 # number of subjects per model

S=1; d=1; R=1; Z=0 # include/exclude measurement parameters

H=1; c=1; G=1; Q=1; P=1; B=0 # include/exclude structural parameters

library(Matrix) # the following code sets up the model in matrix form, see manual

# (fi=fixed values, fr=free estimates, in=starting values, lb=lower bounds, ub=upper bounds)

Sfi=matrix(0,ny,ne)

Sfr=matrix(c(c(1:3),rep(0,6),c(4:6)),ny,ne); n=nnzero(Sfr)

Sin=rep(.5,n); Slb=rep(-100,n);Sub=rep(100,n)

dfi=matrix(0,ny,1)

dfr=matrix(c(20:25),ny,1); n=nnzero(dfr)

din=rep(3,n); dlb=rep(-100,n); dub=rep(100,n)

Rfi=matrix(0,ny,ny)

Rfr=diag(c(30:35)); n=nnzero(Rfr)

Rin=rep(.5,n); Rlb=rep(.000001,n); Rub=rep(100,n)

#Zfi

#Zfr

#Zin;Zlb;Zub

Hfi=matrix(0,ne,ne)

Hfr=matrix(c(40:43),ne,ne);n=nnzero(Hfr)

Hin=rep(.2,n); Hlb=rep(-100,n); Hub=rep(100,n)

cfi=matrix(0,ne,1)

cfr=cfi; n=nnzero(cfr) # scaling contraints

#cin=rep(0,n); clb=rep(-100,n); cub=rep(100,n)

Gfi=diag(ne)

Gfr=matrix(0,ne,ne)

Qfi=diag(ne) # scaling constraints

Qfr=matrix(0,ne,ne);Qfr[lower.tri(Qfr)]<-c(70);n=nnzero(Qfr)

Qin=rep(.4,n); Qlb=rep(-100,n); Qub=rep(100,n)

#Bfi

#Bfr

#Bin;Blb;Bub

Pfi=diag(10,ne);Pfi[lower.tri(Pfi)]<-0

Pfr=matrix(0,ne,ne)

# IF you want to run MKF

run.mkf=0 # runs MKF if and only if run.mkf=1

# IF you want to read in the MKF-output-files (parameter estimates, standard errors etc.) and output a file

read.output=0 # reads in output-files if and only if read=1

npars=23 # number of freely estimated parameters in the model

name.pars=c("S11","S21","S31","S42","S52","S62","d1","d2","d3","d4","d5","d6","R11","R22","R33","R44","R55","R66","H11","H21","H12","H22","Q12")

# names/labels of freely estimated parameters in the model; order must correspond to numerical order of parameters in input-creation-section

# table columns in this order: parameter label, parameter estimate, standard error, t-value, LogLikelihood, AIC (cf. Akaike,1974), BIC (cf. Schwartz,1978), nt, g

# NOW SIMPLY EXECUTE THE FOLLOWING CODE!

#---------------------------------------------------------------------------------------------------------------------------------------------------------

# write data file for mkfm (see manual)

#------------------------------------------------------------------------------

if (write.data==1) {

setwd(path1)

write(nt, file=name.df, nc=1,append=F)

write(t(cvec), file=name.df, nc=ne, append=T)

write(t(data_mkf), file=name.df, nc=ny,append=T)

write("\n", file=name.df, nc=ny,append=T)

}

# write input-files

#---------------------------------------------------------------------------------------------------

if (write.input==1){

setwd(path1)

name001=paste0(name.inf,".txt")

write("title", file=name001, append=F);write("\n", file=name001, append=T)

write(paste0("nm=",ngroup," se=yes"), file=name001, append=T)

write(paste0("mo=",nm," ny=",ny, " ne=",ne," nx=",nx), file=name001, append=T)

write(paste0("df=",name.df," rf=yes"," ns=",ns," mi=-999"), file=name001, append=T)

write(paste0("S=",S," d=",d," R=",R," Z=",Z," H=",H," c=",c," G=",G," Q=",Q," P=",P," B=",B),file=name001, append=T); write("\n", file=name001, append=T)

if(S==1){

write("S fi", file=name001, append=T);write(t(Sfi), file=name001, nc=ncol(Sfi),append=T);write("\n", file=name001, append=T)

write("S fr", file=name001, append=T);write(t(Sfr), file=name001, nc=ncol(Sfi),append=T);write("\n", file=name001, append=T)}

if(d==1){

write("d fi", file=name001, append=T);write(dfi, file=name001, nc=nrow(dfi),append=T);write("\n", file=name001, append=T)

write("d fr", file=name001, append=T);write(dfr, file=name001, nc=nrow(dfi),append=T);write("\n", file=name001, append=T)}

if(R==1){

write("R fi", file=name001, append=T);for (i in (1:ncol(Rfi))) {write(t(Rfi[i,(1:(ncol(Rfi)-(ncol(Rfi)-i)))]), file=name001, nc=(ncol(Rfi)-(ncol(Rfi)-i)),append=T)} ;write("\n", file=name001, append=T)

write("R fr", file=name001, append=T);for (i in (1:ncol(Rfi))) {write(t(Rfr[i,(1:(ncol(Rfi)-(ncol(Rfi)-i)))]), file=name001, nc=(ncol(Rfi)-(ncol(Rfi)-i)),append=T)} ;write("\n", file=name001, append=T) }

if(Z==1){

write("Z fi", file=name001, append=T);write(t(Zfi), file=name001, nc=ncol(Zfi),append=T);write("\n", file=name001, append=T)

write("Z fr", file=name001, append=T);write(t(Zfr), file=name001, nc=ncol(Zfi),append=T);write("\n", file=name001, append=T)}

if(H==1){

write("H fi", file=name001, append=T);write(t(Hfi), file=name001, nc=ncol(Hfi),append=T);write("\n", file=name001, append=T)

write("H fr", file=name001, append=T);write(t(Hfr), file=name001, nc=ncol(Hfi),append=T);write("\n", file=name001, append=T)}

if(c==1){

write("c fi", file=name001, append=T);write(cfi, file=name001, nc=nrow(cfi),append=T);write("\n", file=name001, append=T)

write("c fr", file=name001, append=T);write(cfr, file=name001, nc=nrow(cfi),append=T);write("\n", file=name001, append=T)}

if(G==1){

write("G fi", file=name001, append=T);write(t(Gfi), file=name001, nc=ncol(Gfi),append=T);write("\n", file=name001, append=T)

write("G fr", file=name001, append=T);write(t(Gfr), file=name001, nc=ncol(Gfi),append=T);write("\n", file=name001, append=T)}

if(Q==1){

write("Q fi", file=name001, append=T);for (i in (1:ncol(Qfi))) {write(t(Qfi[i,(1:(ncol(Qfi)-(ncol(Qfi)-i)))]), file=name001, nc=(ncol(Qfi)-(ncol(Qfi)-i)),append=T)} ;write("\n", file=name001, append=T)

write("Q fr", file=name001, append=T);for (i in (1:ncol(Qfi))) {write(t(Qfr[i,(1:(ncol(Qfi)-(ncol(Qfi)-i)))]), file=name001, nc=(ncol(Qfi)-(ncol(Qfi)-i)),append=T)} ;write("\n", file=name001, append=T) }

if(B==1){

write("B fi", file=name001, append=T);write(t(Bfi), file=name001, nc=ncol(Bfi),append=T);write("\n", file=name001, append=T)

write("B fr", file=name001, append=T);write(t(Bfr), file=name001, nc=ncol(Bfi),append=T);write("\n", file=name001, append=T)}

if(P==1){

write("P fi", file=name001, append=T);for (i in (1:ncol(Pfi))) {write(t(Pfi[i,(1:(ncol(Pfi)-(ncol(Pfi)-i)))]), file=name001, nc=(ncol(Pfi)-(ncol(Pfi)-i)),append=T)} ;write("\n", file=name001, append=T)

write("P fr", file=name001, append=T);for (i in (1:ncol(Pfi))) {write(t(Pfr[i,(1:(ncol(Pfi)-(ncol(Pfi)-i)))]), file=name001, nc=(ncol(Pfi)-(ncol(Pfi)-i)),append=T)} ;write("\n", file=name001, append=T) }

write("st", file=name001, append=T)

if(S==1){if(nnzero(Sfr)>0){write(Sin,file=name001, append=T)}}

if(d==1){if(nnzero(dfr)>0){write(din,file=name001, append=T)}}

if(R==1){if(nnzero(Rfr)>0){write(Rin,file=name001, append=T)}}

if(Z==1){if(nnzero(Zfr)>0){write(Zin,file=name001, append=T)}}

if(H==1){if(nnzero(Hfr)>0){write(Hin,file=name001, append=T)}}

if(c==1){if(nnzero(cfr)>0){write(cin,file=name001, append=T)}}

if(G==1){if(nnzero(Gfr)>0){write(Gin,file=name001, append=T)}}

if(Q==1){if(nnzero(Qfr)>0){write(Qin,file=name001, append=T)}}

if(B==1){if(nnzero(Bfr)>0){write(Bin,file=name001, append=T)}}; write("\n", file=name001, append=T)

write("lb", file=name001, append=T)

if(S==1){if(nnzero(Sfr)>0){write(Slb,file=name001, append=T)}}

if(d==1){if(nnzero(dfr)>0){write(dlb,file=name001, append=T)}}

if(R==1){if(nnzero(Rfr)>0){write(Rlb,file=name001, append=T)}}

if(Z==1){if(nnzero(Zfr)>0){write(Zlb,file=name001, append=T)}}

if(H==1){if(nnzero(Hfr)>0){write(Hlb,file=name001, append=T)}}

if(c==1){if(nnzero(cfr)>0){write(clb,file=name001, append=T)}}

if(G==1){if(nnzero(Gfr)>0){write(Glb,file=name001, append=T)}}

if(Q==1){if(nnzero(Qfr)>0){write(Qlb,file=name001, append=T)}}

if(B==1){if(nnzero(Bfr)>0){write(Blb,file=name001, append=T)}}; write("\n", file=name001, append=T)

write("ub", file=name001, append=T)

if(S==1){if(nnzero(Sfr)>0){write(Sub,file=name001, append=T)}}

if(d==1){if(nnzero(dfr)>0){write(dub,file=name001, append=T)}}

if(R==1){if(nnzero(Rfr)>0){write(Rub,file=name001, append=T)}}

if(Z==1){if(nnzero(Zfr)>0){write(Zub,file=name001, append=T)}}

if(H==1){if(nnzero(Hfr)>0){write(Hub,file=name001, append=T)}}

if(c==1){if(nnzero(cfr)>0){write(cub,file=name001, append=T)}}

if(G==1){if(nnzero(Gfr)>0){write(Gub,file=name001, append=T)}}

if(Q==1){if(nnzero(Qfr)>0){write(Qub,file=name001, append=T)}}

if(B==1){if(nnzero(Bfr)>0){write(Bub,file=name001, append=T)}}; write("\n", file=name001, append=T) }

# run mkfm

#------------------------------------------------------------------------------

if (run.mkf==1){

setwd(path1)

command<-paste("mkfm6-1<",name.inf,".txt",">",name.ouf,".txt",sep="")

shell(command, wait=T) # run mkfm and halt execution of R-code until it's finished

}

# read output-files & write summary table

#---------------------------------------------------------------------------------------------------

if (read.output==1) {

setwd(path1)

output<-matrix(0,nrow=npars,ncol=9)

colnames(output)<-c("parameter","estimate","se","t","LogL","AIC","BIC","nt","g")

output[,1]<-as.matrix(name.pars)

out<-read.table(paste(name.ouf,".txt",sep=""),fill=TRUE)

out<-as.matrix(out)

line=grep('ML',out)

out1<-out[(line+1):(line+npars),]

output[,2]<-out1[,3]

output[,3]<-out1[,7]

output[,4]<-out1[,9]

output[,9]<-out1[,5]

line=grep('Logl',out)

output[,5]<-out[line,2]

output[,6]<-((-2)*as.numeric(out[line,2])+2*npars)

output[,7]<-((-2)*as.numeric(out[line,2])+npars*log(nt))

output[,8]<-nt

setwd(path2)

write.table(output,file=paste(name.ouf,"_summary.txt",sep=""),col.names=T,row.names=F) } }

**MKF Input and Output**

All MKF in- and output files available

**Individual models**

| **Model (cf. table 1)** | **Subject** | **Inputfile / outputfile** |
| --- | --- | --- |
| VAR(0) | 7 | inf2a0p71.txt / ouf2a0p71.txt |
| VAR(1) |  | inf2a1p71.txt / ouf2a1p71.txt |
| *VAR(1)** |  | inf2a1p72.txt / ouf2a1p72.txt |
| VAR(2) |  | inf2a2p71.txt / ouf2a2p71.txt |
| *VAR(0)* | 13 | inf2a0p131.txt / ouf2a0p131.txt |
| VAR(1) |  | inf2a1p131.txt / ouf2a1p131.txt |
| VAR(2) |  | inf2a2p131.txt / ouf2a2p131.txt |
| VAR(0) | 22 | inf2a0p221.txt / ouf2a0p221.txt |
| VAR(0)* |  | inf2a0p222.txt / ouf2a0p222.txt |
| VAR(1) |  | inf2a1p221.txt / ouf2a1p221.txt |
| VAR(1)* |  | inf2a1p222.txt / ouf2a1p222.txt |
| *VAR(2)* |  | inf2a2p221.txt / ouf2a2p221.txt |
| VAR(2)* |  | inf2a2p222.txt / ouf2a2p222.txt |

**Multi-group models**

| **Model (cf. table 2)** | **Subjects** | **Inputfile / outputfile** |
| --- | --- | --- |
| Configural  invariance | 7 – 13 | inpp713M1.txt / oupp713M1.txt |
| *Weak FI (*$\boldsymbol{\Lambda}$ *invariant)* |  | inpp713M2.txt / oupp713M2.txt |
| Strong FI ($\boldsymbol{\Lambda}$**,** $\boldsymbol{\tau}$ invariant) |  | inpp713M3.txt / oupp713M3.txt |
| Strict FI ($\boldsymbol{\Lambda}$**,** $\boldsymbol{\tau}$**,** $\boldsymbol{\Theta}$ invariant) |  | inpp713M4.txt / oupp713M4.txt |
| Configural  invariance | 7 – 22 | inpp722M1.txt / oupp722M1.txt |
| *Weak FI (*$\boldsymbol{\Lambda}$ *invariant)* |  | inpp722M2.txt / oupp722M2.txt |
| Strong FI ($\boldsymbol{\Lambda}$**,** $\boldsymbol{\tau}$ invariant) |  | inpp722M3.txt / oupp722M3.txt |
| Strict FI ($\boldsymbol{\Lambda}$**,** $\boldsymbol{\tau}$**,** $\boldsymbol{\Theta}$ invariant) |  | inpp722M4.txt / oupp722M4.txt |
| Configural  invariance | 13 – 22 | Inpp1322M1.txt / oupp1322M1.txt |
| *Weak FI (*$\boldsymbol{\Lambda}$ *invariant)* |  | Inpp1322M2.txt / oupp1322M2.txt |
| Strong FI ($\boldsymbol{\Lambda}$**,** $\boldsymbol{\tau}$ invariant) |  | Inpp1322M3.txt / oupp1322M3.txt |
| Strict FI ($\boldsymbol{\Lambda}$**,** $\boldsymbol{\tau}$**,** $\boldsymbol{\Theta}$ invariant) |  | Inpp1322M3.txt / oupp1322M3.txt |

**Model including a fixed regressor**

| **Model (cf. table 3)** | **Subject** | **Inputfile/outputfile** |
| --- | --- | --- |
| $\boldsymbol{y}$, $\boldsymbol{\eta}$ on $\boldsymbol{x}$ | 7 | inf2a1xp71.txt / ouf2a1xp71.txt |
| $\boldsymbol{\eta}$ on $\boldsymbol{x}$ |  | inf2a1xp75.txt / ouf2a1xp75.txt |
| $\boldsymbol{y}(a)$, $\boldsymbol{\eta}(a)$ on $\boldsymbol{x}$ |  | inf2a1xp73.txt / ouf2a1xp73.txt |
| $\boldsymbol{\eta}(a)$ on $\boldsymbol{x}$ |  | inf2a1xp74.txt / ouf2a1xp74.txt |
