# Supplementary material for: Measurement invariance within and between individuals: a distinct problem in testing the equivalence of intra- and inter-individual model structures
Source: Front Psychol. 2014 Sep 19;5:883. doi: 10.3389/fpsyg.2014.00883 (PMC4193237; doi:10.3389/fpsyg.2014.00883)
Supplement: Supplementary file 1 [file DataSheet1.ZIP › MIadolf_supplements.pdf]

Supplementary Material

Measurement invariance within and between individuals: A distinct problem in testing the equivalence of intra- and inter-individual model structures.

Janne Adolf[1]*, Noémi Schuurman[2], Peter Borkenau[3], Denny Borsboom[4] and Conor Dolan[5]

[1]Center for Lifespan Psychology, Max Planck Institute for Human Development, Berlin, Germany
[2]Department of Methodology and Statistics, Faculty of Social and Behavioral Sciences, Utrecht University, Utrecht, The Netherlands
[3]Personality and Diagnostics Group, Department of Psychology, Faculty of Philosophy I, Martin-Luther-University Halle-Wittenberg, Halle, Germany
[4]Psychological Methods Group, Department of Psychology, Faculty of Social and Behavioral Sciences, University of Amsterdam, Amsterdam, The Netherlands
[5]Department of Biological Psychology, Faculty of Psychology and Education, Free University of Amsterdam, Amsterdam, The Netherlands

* **Correspondence**: Janne Adolf, Center for Lifespan Psychology, Max Planck Institute for Human Development, Lentzeallee 94, 14195 Berlin, Germany.
adolf@mpib-berlin.mpg.de

Highlights

The following points summarize the specific contribution of our paper to the special issue on measurement invariance

- We discuss how the concept of measurement invariance can generally and operationally be applied at the intra-individual and the inter-individual level of analysis.
- We show that measurement invariance holding simultaneously over time and subject can be interpreted as constituting a mode of structural equivalence between the intra- and the inter-individual level of analysis that is distinct from full structural equivalence.
- We illustrate how measurement invariance can be tested over subjects and over time in the context of (time-invariant) multi-subject time series models in state-space format.
- We strengthen the view that heterogeneity between and within subjects is a measurement problem that can be addressed in the context of latent variable modeling.

Supplementary figures

The following supplementary figures show the raw time series and frequency distributions of all individual’s responses over time.

Supplementary figure 1. Responses to extraversion marker items as provided by subject 7.

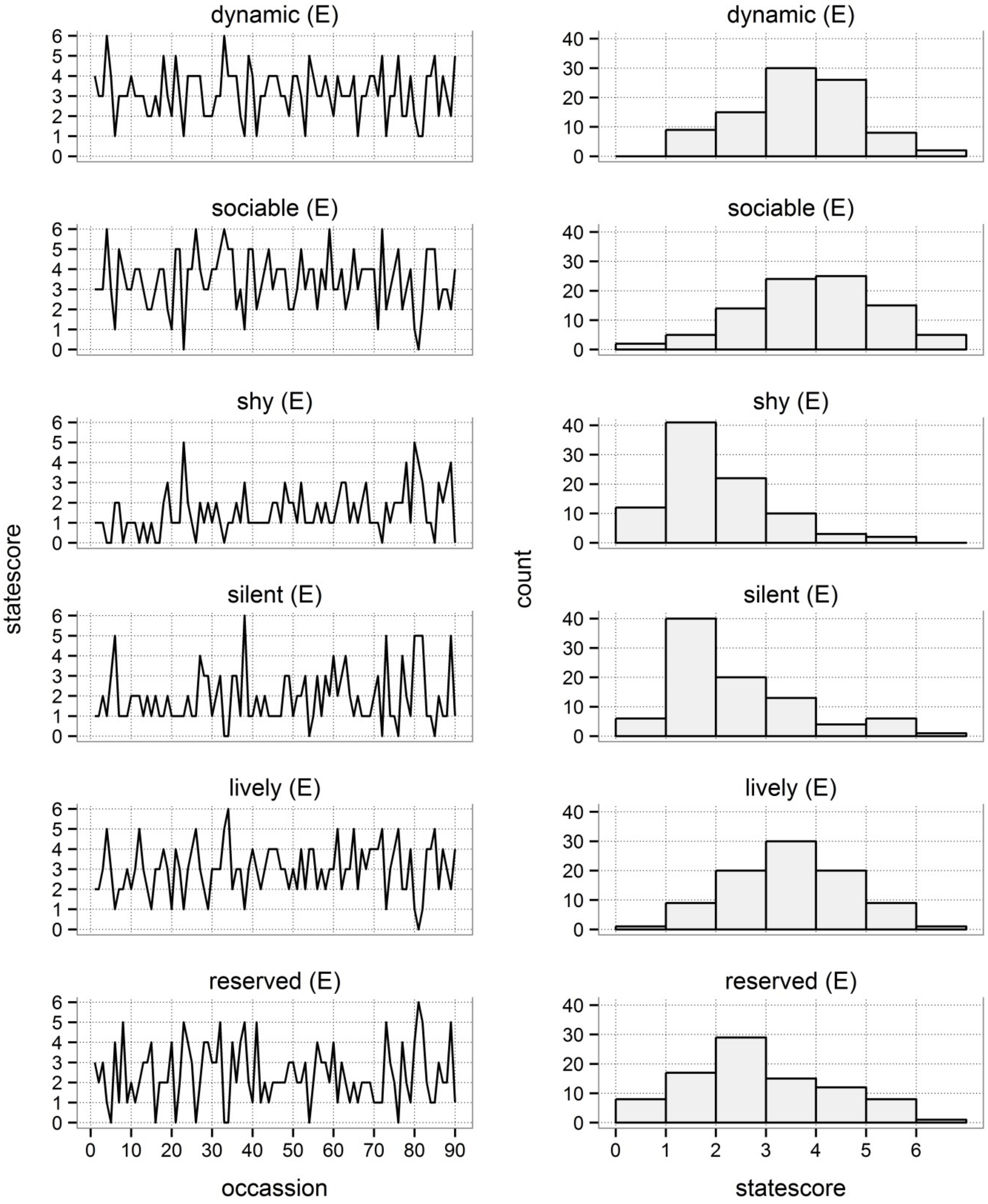

Supplementary figure 2. Responses to agreeableness marker items as provided by subject 7.

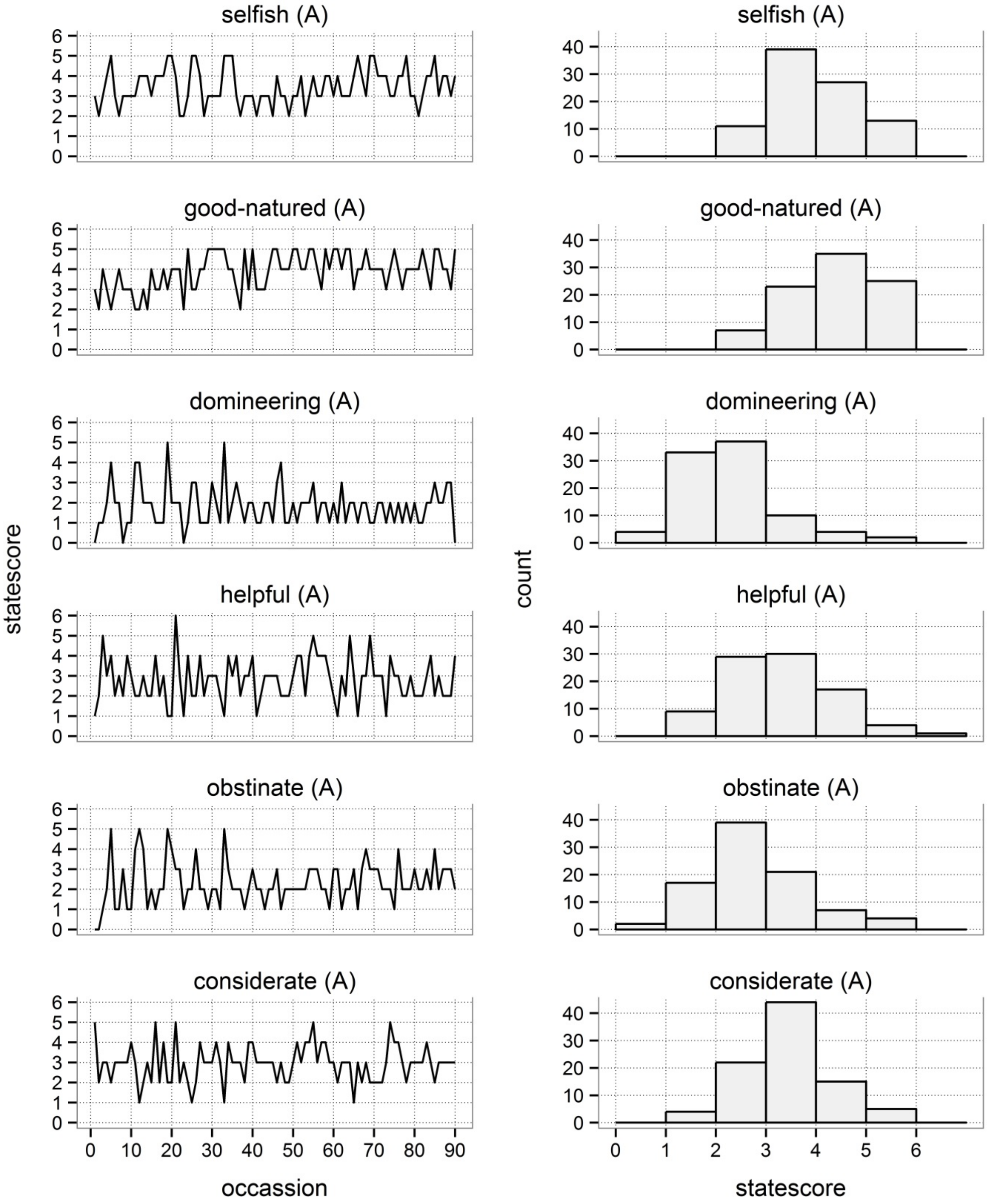

Supplementary figure 3. Responses to extraversion marker items as provided by subject 13.

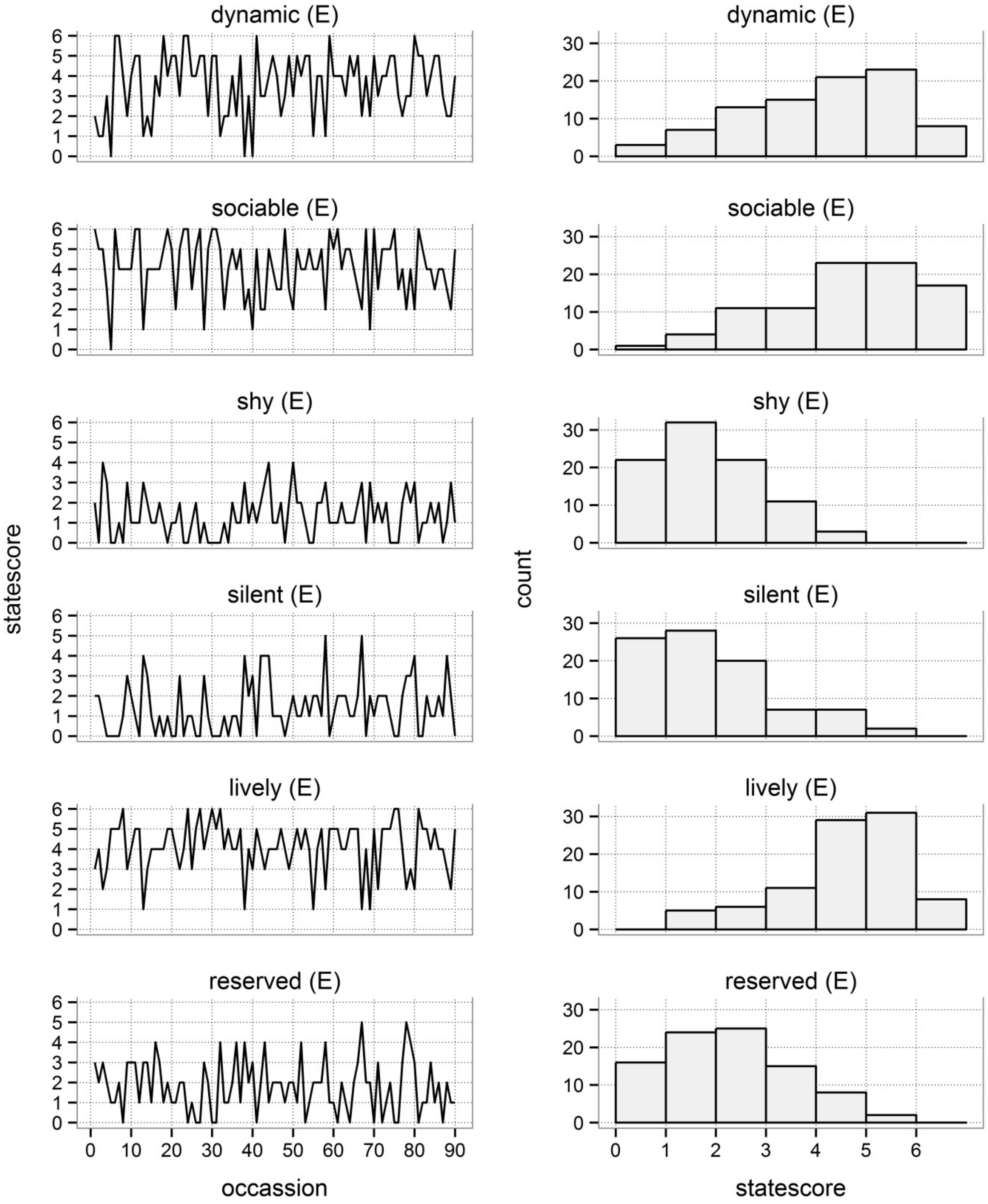

Supplementary figure 4. Responses to agreeableness marker items as provided by subject 13.

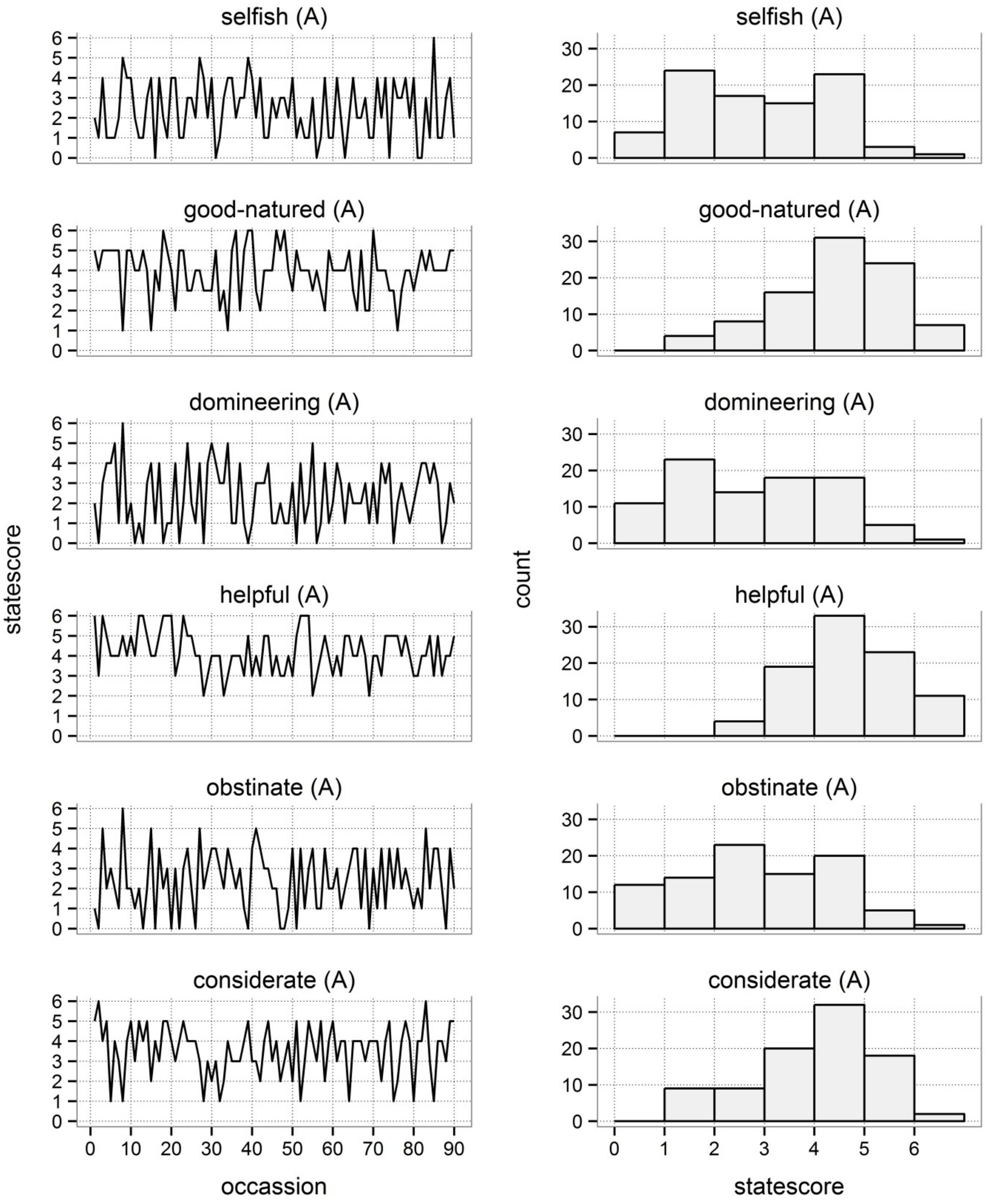

Supplementary figure 5. Responses to extraversion marker items as provided by subject 22.

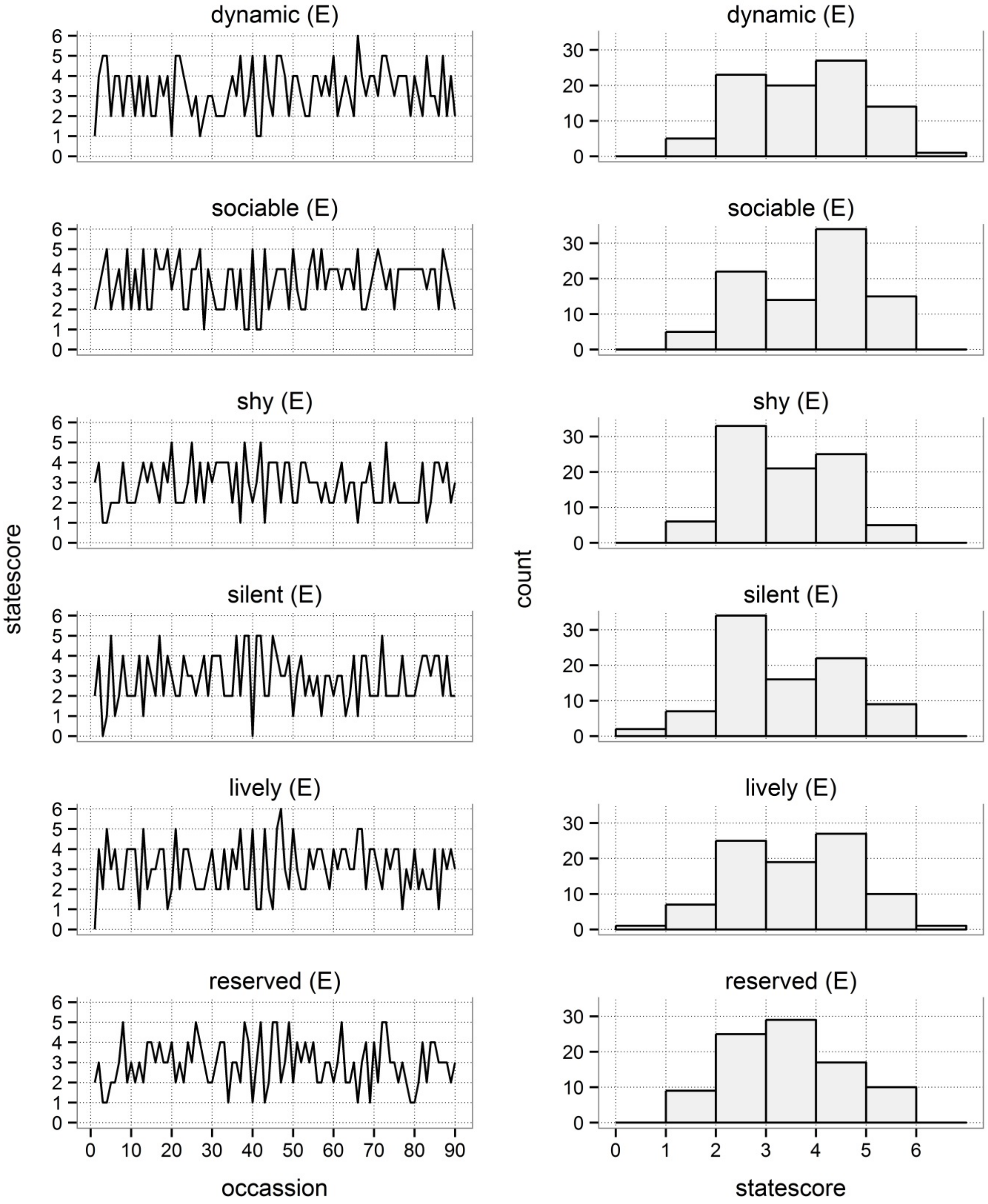

Supplementary figure 6. Responses to agreeableness marker items as provided by subject 22.

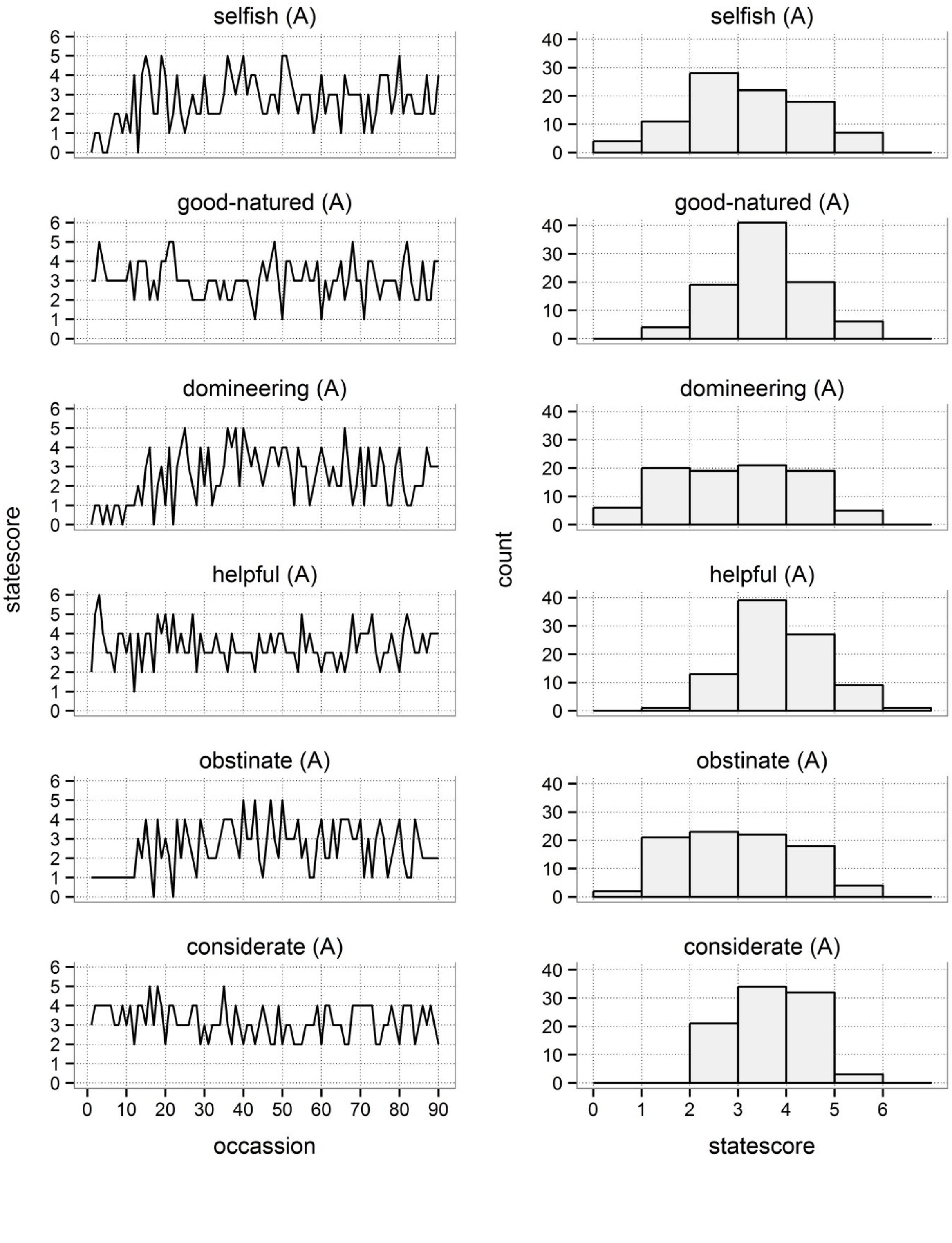

Data

Data files in MKF-format available for subjects 7, 13, and 22 and selected items.

MKF via R

The following R-code can be used to set up data and input files for MKF from R, execute MKF under R, and read MKF output files into R (also available file MKFviaR.R).

```
#*********************************#
# MKFM6-1-WRAPPER-LIKE-CODE FOR R #
#*********************************#


# INFORMATION TO BE SPECIFIED
#-----------------------------------------------------------------------------

path1<-"C:/User/folderA"   # working directory;
      # must contain "mkfm6-1.exe", datafiles (in suitable format, see manual), input-files (in suitable format, see manual or use this script)
path2<-"C:/User/folderB"   # directory to save the results-table in

nt=100   # number of time points / length of time series
ny=6     # number of manifest variables
ne=2           # number of latent factors
nx=0     # number of time-varying covariates

name.inf="inp1"   # name of mkfm-inputfile; input-files can be created using this code (otherwise to be prepared manually, see manual)
name.ouf="oup1"   # name of mkfm-outputfile; will be created by this code)
name.df="dp1"     # name of data file


# IF you have a data-object in R and want to write data-files for mkfm

write.data=0                              # writes data-files if and only if write=1
data_mkf<-data.p1                        # R-object containing the data in in long-format (observations in rows, variables in columns)
cvec=matrix(0,ne,1)   # vector containing starting values for the latent variables (expected values at t0) ! WRAPPER NEEDS TO BE EXTEND-
ED FOR AR(>1)


# IF you want to set-up the input-files from R; DEFAULTS: missings are expected to be -999, se=yes, rf=yes, it=1000 (see manual)

write.input=0       # sets up input-files if and only if write=1
ngroup=1           # number of groups ! WRAPPER NEEDS ADJUSTMENT FOR NGROUP>1
nm=1               # number of model/index of group model
ns=1              # number of subjects per model
S=1; d=1; R=1; Z=0          # include/exclude measurement parameters
H=1; c=1; G=1; Q=1; P=1; B=0  # include/exclude structural parameters
library(Matrix)            # the following code sets up the model in matrix form, see manual
                          # (fi=fixed values, fr=free estimates, in=starting values, lb=lower bounds, ub=upper bounds)
Sfi=matrix(0,ny,ne)
Sfr=matrix(c(c(1:3),rep(0,6),c(4:6)),ny,ne); n=nnzero(Sfr)
Sin=rep(.5,n); Slb=rep(-100,n);Sub=rep(100,n)

dfi=matrix(0,ny,1)
dfr=matrix(c(20:25),ny,1); n=nnzero(dfr)
din=rep(3,n); dlb=rep(-100,n); dub=rep(100,n)

Rfi=matrix(0,ny,ny)
Rfr=diag(c(30:35)); n=nnzero(Rfr)
Rin=rep(.5,n); Rlb=rep(.000001,n); Rub=rep(100,n)

#Zfi
#Zfr
#Zin;Zlb;Zub

Hfi=matrix(0,ne,ne)
Hfr=matrix(c(40:43),ne,ne);n=nnzero(Hfr)
Hin=rep(.2,n); Hlb=rep(-100,n); Hub=rep(100,n)
```

```
cfi=matrix(0,ne,1)
cfr=cfi; n=nnzero(cfr) # scaling contraints
#cin=rep(0,n); clb=rep(-100,n); cub=rep(100,n)

Gfi=diag(ne)
Gfr=matrix(0,ne,ne)

Qfi=diag(ne) # scaling constraints
Qfr=matrix(0,ne,ne);Qfr[lower.tri(Qfr)]<-c(70);n=nnzero(Qfr)
Qin=rep(.4,n); Qlb=rep(-100,n); Qub=rep(100,n)

#Bfi
#Bfr
#Bin;Blb;Bub

Pfi=diag(10,ne);Pfi[lower.tri(Pfi)]<-0
Pfr=matrix(0,ne,ne)


# IF you want to run MKF
run.mkf=0        # runs MKF if and only if run.mkf=1


# IF you want to read in the MKF-output-files (parameter estimates, standard errors etc.) and output a file
read.output=0                                        # reads in output-files if and only if read=1
npars=23                                             # number of freely estimated parameters in the model
name.pars=c("S11","S21","S31","S42","S52","S62","d1","d2","d3","d4","d5","d6","R11","R22","R33","R44","R55","R66","H11","H21","H12
","H22","Q12")
        # names/labels of freely estimated parameters in the model; order must correspond to numerical order of parameters in input-
creation-section
        # table columns in this order: parameter label, parameter estimate, standard error, t-value, LogLikelihood, AIC (cf. Akaike,1974), BIC
(cf. Schwartz,1978), nt, g



#  NOW SIMPLY EXECUTE THE FOLLOWING CODE!
#--------------------------------------------------------------------------------------------------------------------------------------------------------

# write data file for mkfm (see manual)
#------------------------------------------------------------------------------

if (write.data==1) {
  setwd(path1)
  write(nt, file=name.df, nc=1,append=F)
  write(t(cvec), file=name.df, nc=ne, append=T)
  write(t(data_mkf), file=name.df, nc=ny,append=T)
  write("\n", file=name.df, nc=ny,append=T)
}

# write input-files
#----------------------------------------------------------------------------------------------------

if (write.input==1){
  setwd(path1)
  name001=paste0(name.inf,".txt")
  write("title", file=name001, append=F);write("\n", file=name001, append=T)
  write(paste0("nm=",ngroup," se=yes"), file=name001, append=T)
  write(paste0("mo=",nm," ny=",ny, " ne=",ne," nx=",nx), file=name001, append=T)
  write(paste0("df=",name.df," rf=yes"," ns=",ns," mi=-999"), file=name001, append=T)
  write(paste0("S=",S," d=",d," R=",R," Z=",Z," H=",H," c=",c," G=",G," Q=",Q," P=",P," B=",B),file=name001, append=T); write("\n",
file=name001, append=T)
  if(S==1){
    write("S fi", file=name001, append=T);write(t(Sfi), file=name001, nc=ncol(Sfi),append=T);write("\n", file=name001, append=T)
    write("S fr", file=name001, append=T);write(t(Sfr), file=name001, nc=ncol(Sfi),append=T);write("\n", file=name001, append=T)}
  if(d==1){
    write("d fi", file=name001, append=T);write(dfi, file=name001, nc=nrow(dfi),append=T);write("\n", file=name001, append=T)
    write("d fr", file=name001, append=T);write(dfr, file=name001, nc=nrow(dfi),append=T);write("\n", file=name001, append=T)}
  if(R==1){
    write("R fi", file=name001, append=T);for (i in (1:ncol(Rfi))) {write(t(Rfi[i,(1:(ncol(Rfi)-(ncol(Rfi)-i)))]), file=name001, nc=(ncol(Rfi)-
(ncol(Rfi)-i)),append=T)} ;write("\n", file=name001, append=T)
```

```
   write("R fr", file=name001, append=T);for (i in (1:ncol(Rfi))) {write(t(Rfr[i,(1:(ncol(Rfi)-(ncol(Rfi)-i)))]), file=name001, nc=(ncol(Rfi)-(ncol(Rfi)-i)),append=T)} ;write("\n", file=name001, append=T) }
  if(Z==1){
   write("Z fi", file=name001, append=T);write(t(Zfi), file=name001, nc=ncol(Zfi),append=T);write("\n", file=name001, append=T)
   write("Z fr", file=name001, append=T);write(t(Zfr), file=name001, nc=ncol(Zfi),append=T);write("\n", file=name001, append=T)}
  if(H==1){
   write("H fi", file=name001, append=T);write(t(Hfi), file=name001, nc=ncol(Hfi),append=T);write("\n", file=name001, append=T)
   write("H fr", file=name001, append=T);write(t(Hfr), file=name001, nc=ncol(Hfi),append=T);write("\n", file=name001, append=T)}
  if(c==1){
   write("c fi", file=name001, append=T);write(cfi, file=name001, nc=nrow(cfi),append=T);write("\n", file=name001, append=T)
   write("c fr", file=name001, append=T);write(cfr, file=name001, nc=nrow(cfi),append=T);write("\n", file=name001, append=T)}
  if(G==1){
   write("G fi", file=name001, append=T);write(t(Gfi), file=name001, nc=ncol(Gfi),append=T);write("\n", file=name001, append=T)
   write("G fr", file=name001, append=T);write(t(Gfr), file=name001, nc=ncol(Gfi),append=T);write("\n", file=name001, append=T)}
  if(Q==1){
   write("Q fi", file=name001, append=T);for (i in (1:ncol(Qfi))) {write(t(Qfi[i,(1:(ncol(Qfi)-(ncol(Qfi)-i)))]), file=name001, nc=(ncol(Qfi)-(ncol(Qfi)-i)),append=T)} ;write("\n", file=name001, append=T)
   write("Q fr", file=name001, append=T);for (i in (1:ncol(Qfi))) {write(t(Qfr[i,(1:(ncol(Qfi)-(ncol(Qfi)-i)))]), file=name001, nc=(ncol(Qfi)-(ncol(Qfi)-i)),append=T)} ;write("\n", file=name001, append=T) }
  if(B==1){
   write("B fi", file=name001, append=T);write(t(Bfi), file=name001, nc=ncol(Bfi),append=T);write("\n", file=name001, append=T)
   write("B fr", file=name001, append=T);write(t(Bfr), file=name001, nc=ncol(Bfi),append=T);write("\n", file=name001, append=T)}
  if(P==1){
   write("P fi", file=name001, append=T);for (i in (1:ncol(Pfi))) {write(t(Pfi[i,(1:(ncol(Pfi)-(ncol(Pfi)-i)))]), file=name001, nc=(ncol(Pfi)-(ncol(Pfi)-i)),append=T)} ;write("\n", file=name001, append=T)
   write("P fr", file=name001, append=T);for (i in (1:ncol(Pfi))) {write(t(Pfr[i,(1:(ncol(Pfi)-(ncol(Pfi)-i)))]), file=name001, nc=(ncol(Pfi)-(ncol(Pfi)-i)),append=T)} ;write("\n", file=name001, append=T) }
  write("st", file=name001, append=T)
  if(S==1){if(nnzero(Sfr)>0){write(Sin,file=name001, append=T)}}
  if(d==1){if(nnzero(dfr)>0){write(din,file=name001, append=T)}}
  if(R==1){if(nnzero(Rfr)>0){write(Rin,file=name001, append=T)}}
  if(Z==1){if(nnzero(Zfr)>0){write(Zin,file=name001, append=T)}}
  if(H==1){if(nnzero(Hfr)>0){write(Hin,file=name001, append=T)}}
  if(c==1){if(nnzero(cfr)>0){write(cin,file=name001, append=T)}}
  if(G==1){if(nnzero(Gfr)>0){write(Gin,file=name001, append=T)}}
  if(Q==1){if(nnzero(Qfr)>0){write(Qin,file=name001, append=T)}}
  if(B==1){if(nnzero(Bfr)>0){write(Bin,file=name001, append=T)}}; write("\n", file=name001, append=T)
  write("lb", file=name001, append=T)
  if(S==1){if(nnzero(Sfr)>0){write(Slb,file=name001, append=T)}}
  if(d==1){if(nnzero(dfr)>0){write(dlb,file=name001, append=T)}}
  if(R==1){if(nnzero(Rfr)>0){write(Rlb,file=name001, append=T)}}
  if(Z==1){if(nnzero(Zfr)>0){write(Zlb,file=name001, append=T)}}
  if(H==1){if(nnzero(Hfr)>0){write(Hlb,file=name001, append=T)}}
  if(c==1){if(nnzero(cfr)>0){write(clb,file=name001, append=T)}}
  if(G==1){if(nnzero(Gfr)>0){write(Glb,file=name001, append=T)}}
  if(Q==1){if(nnzero(Qfr)>0){write(Qlb,file=name001, append=T)}}
  if(B==1){if(nnzero(Bfr)>0){write(Blb,file=name001, append=T)}}; write("\n", file=name001, append=T)
  write("ub", file=name001, append=T)
  if(S==1){if(nnzero(Sfr)>0){write(Sub,file=name001, append=T)}}
  if(d==1){if(nnzero(dfr)>0){write(dub,file=name001, append=T)}}
  if(R==1){if(nnzero(Rfr)>0){write(Rub,file=name001, append=T)}}
  if(Z==1){if(nnzero(Zfr)>0){write(Zub,file=name001, append=T)}}
  if(H==1){if(nnzero(Hfr)>0){write(Hub,file=name001, append=T)}}
  if(c==1){if(nnzero(cfr)>0){write(cub,file=name001, append=T)}}
  if(G==1){if(nnzero(Gfr)>0){write(Gub,file=name001, append=T)}}
  if(Q==1){if(nnzero(Qfr)>0){write(Qub,file=name001, append=T)}}
  if(B==1){if(nnzero(Bfr)>0){write(Bub,file=name001, append=T)}}; write("\n", file=name001, append=T) }

# run mkfm
#-------------------------------------------------------------------------------
if (run.mkf==1){
  setwd(path1)
  command<-paste("mkfm6-1<",name.inf,".txt",">",name.ouf,".txt",sep="")
  shell(command, wait=T)                        # run mkfm and halt execution of R-code until it's finished

}

# read output-files & write summary table
#----------------------------------------------------------------------------------------------------
if (read.output==1) {
  setwd(path1)
```

```
output<-matrix(0,nrow=npars,ncol=9)
colnames(output)<-c("parameter","estimate","se","t","LogL","AIC","BIC","nt","g")
output[,1]<-as.matrix(name.pars)
out<-read.table(paste(name.ouf,".txt",sep=""),fill=TRUE)
out<-as.matrix(out)
line=grep('ML',out)
out1<-out[(line+1):(line+npars),]
output[,2]<-out1[,3]
output[,3]<-out1[,7]
output[,4]<-out1[,9]
output[,9]<-out1[,5]
line=grep('Logl',out)
output[,5]<-out[line,2]
output[,6]<-((-2)*as.numeric(out[line,2])+2*npars)
output[,7]<-((-2)*as.numeric(out[line,2])+npars*log(nt))
output[,8]<-nt
setwd(path2)
write.table(output,file=paste(name.ouf,"_summary.txt",sep=""),col.names=T,row.names=F) } }
```

MKF Input and Output

All MKF in- and output files available

Individual models

Model (cf. table 1)	Subject	Inputfile / outputfile
VAR(0)	7	inf2a0p71.txt / ouf2a0p71.txt
VAR(1)		inf2a1p71.txt / ouf2a1p71.txt
*VAR(1)**		inf2a1p72.txt / ouf2a1p72.txt
VAR(2)		inf2a2p71.txt / ouf2a2p71.txt
VAR(0)	13	inf2a0p131.txt / ouf2a0p131.txt
VAR(1)		inf2a1p131.txt / ouf2a1p131.txt
VAR(2)		inf2a2p131.txt / ouf2a2p131.txt
VAR(0)	22	inf2a0p221.txt / ouf2a0p221.txt
VAR(0)*		inf2a0p222.txt / ouf2a0p222.txt
VAR(1)		inf2a1p221.txt / ouf2a1p221.txt
VAR(1)*		inf2a1p222.txt / ouf2a1p222.txt
VAR(2)		inf2a2p221.txt / ouf2a2p221.txt
VAR(2)*		inf2a2p222.txt / ouf2a2p222.txt

Multi-group models

Model (cf. table 2)	Subjects	Inputfile / outputfile
Configural invariance	7 – 13	inpp713M1.txt / oupp713M1.txt
Weak FI (Λ invariant)		inpp713M2.txt / oupp713M2.txt
Strong FI		inpp713M3.txt / oupp713M3.txt

($\boldsymbol{\Lambda}$, $\boldsymbol{\tau}$ invariant)		
Strict FI<br>($\boldsymbol{\Lambda}$, $\boldsymbol{\tau}$, $\boldsymbol{\Theta}$ invariant)		inpp713M4.txt / oupp713M4.txt
Configural invariance	7 – 22	inpp722M1.txt / oupp722M1.txt
Weak FI<br>*($\boldsymbol{\Lambda}$ invariant)*		inpp722M2.txt / oupp722M2.txt
Strong FI<br>($\boldsymbol{\Lambda}$, $\boldsymbol{\tau}$ invariant)		inpp722M3.txt / oupp722M3.txt
Strict FI<br>($\boldsymbol{\Lambda}$, $\boldsymbol{\tau}$, $\boldsymbol{\Theta}$ invariant)		inpp722M4.txt / oupp722M4.txt
Configural invariance	13 – 22	Inpp1322M1.txt / oupp1322M1.txt
Weak FI<br>*($\boldsymbol{\Lambda}$ invariant)*		Inpp1322M2.txt / oupp1322M2.txt
Strong FI<br>($\boldsymbol{\Lambda}$, $\boldsymbol{\tau}$ invariant)		Inpp1322M3.txt / oupp1322M3.txt
Strict FI<br>($\boldsymbol{\Lambda}$, $\boldsymbol{\tau}$, $\boldsymbol{\Theta}$ invariant)		Inpp1322M3.txt / oupp1322M3.txt

Model including a fixed regressor

**Model (cf. table 3)**	**Subject**	**Inputfile/outputfile**
$\boldsymbol{y}$, $\boldsymbol{\eta}$ on $\boldsymbol{x}$	7	inf2a1xp71.txt / ouf2a1xp71.txt
$\boldsymbol{\eta}$ on $\boldsymbol{x}$		inf2a1xp75.txt / ouf2a1xp75.txt
$\boldsymbol{y}(a)$, $\boldsymbol{\eta}(a)$ on $\boldsymbol{x}$		inf2a1xp73.txt / ouf2a1xp73.txt
$\boldsymbol{\eta}(a)$ on $\boldsymbol{x}$		inf2a1xp74.txt / ouf2a1xp74.txt
